# Behavioral phenotyping of cancer pain in domesticated cats with naturally occurring squamous cell carcinoma of the tongue: initial validation studies provide evidence for regional and widespread algoplasticity

**DOI:** 10.7717/peerj.11984

**Published:** 2021-08-16

**Authors:** Yen-Hao Erik Lai, B. Duncan X. Lascelles, Michael W. Nolan

**Affiliations:** 1 Comparative Medicine Institute, North Carolina State University, Raleigh, NC, United States of America; 2 Department of Clinical Sciences, College of Veterinary Medicine, North Carolina State University, Raleigh, NC, United States of America; 3 Comparative Pain Research and Education Center, North Carolina State University, Raleigh, NC, United States of America

**Keywords:** Comparative oncology, Comparative pain research, Animal models, Translational research, Feline

## Abstract

Feline oral squamous cell carcinoma (FOSCC) is a common and naturally occurring condition that recapitulates many features of human head and neck cancer (HNC). In both species, there is need for improved strategies to reduce pain caused by HNC and its treatment. Research to benefit both species could be conducted using pet cats as a comparative model, but this prospect is limited by lack of validated methods for quantifying FOSCC-associated pain. A prospective non-randomized pilot study was performed for initial validation of: (1) a pet owner administered quality of life questionnaire and visual assessment scoring tool (FORQ/CLIENT); (2) a clinician assessment questionnaire (UFEPS/VET); (3) electronic von Frey testing [EVF]; and (4) Cochet-Bonnet (COBO) aesthesiometry. To assess intra-rater reliability, discriminatory ability, and responsiveness of each assay, 6 cats with sublingual SCC and 16 healthy control cats were enrolled. The intra-rater reliability was moderate-to-good for the clinical metrology instruments and EVF (intraclass correlation coefficient [ICC] ≥ 0.68), but poor for COBO (ICC = 0.21). FORQ/CLIENT scores were higher (worse quality of life) in FOSCC cats vs healthy controls. The internal reliability of FORQ/CLIENT scoring was high (Cronbach *α* = 0.92); sensitivity and specificity were excellent (100% when using cut-offs determined using receiver operating characteristic [ROC] curves). For the FORQ/CLIENT, there was strong and inverse correlation between scores from the questions and visual assessment (*r* =  − 0.77, *r*^2^ = 0.6, *P* < 0.0001). For the UFEPS/VET, Cronbach’s *α* was 0.74 (high reliability). Sensitivity and specificity were 100% and 94%, respectively, when using a cut-off score (3.5) based on ROC curves (Youden index of 0.94). Total UFEPS/VET scores were positively correlated with FORQ/CLIENT scores (*r*^2^ = 0.72, *P* < 0.0001). Sensitivity of EVF and COBO ranged from 83 to 100% and specificity ranged from 56 to 94%. Cats with cancer were more sensitive around the face (lower response thresholds) and on the cornea (longer filament lengths) than control animals (*P* < 0.03). Reduced pressure response thresholds were also observed at a distant site (*P* = 0.0002) in cancer cats. After giving buprenorphine, EVF pressure response thresholds increased (*P* = 0.04) near the mandible of cats with OSCC; the length of filament required to elicit a response in the COBO assay also improved (shortened; *P* = 0.017). Based on these preliminary assessments, the assays described herein had reasonable inter-rater reliability, and they were able to both discriminate between cats with and without oral cancer, and respond in a predictable manner to analgesic therapy. In cats with tongue cancer, there was evidence for regional peripheral sensitization, and widespread somatosensory sensitization. These results provide a basis for multi-dimensional assessments of pain and sensitivity in cats with oral SCC.

## Introduction

Pain is a common symptom of human head and neck cancer (HNC). Approximately three in five HNC patients report pain (Macfarlane et al., 2012). Effective pain relief is not achieved with currently available analgesics (Portenoy, 2011; Ripamonti et al., 2012), and the commonly used opioids are addictive, cause significant side effects, and have abuse potential (McNicol et al., 2003). The need for improved HNC analgesics is underscored by the fact that each year, HNC is diagnosed in more than 650,000 people worldwide (Bray et al., 2018). Unfortunately, translational pain research based solely on induced rodent models and then human clinical trials is not efficiently producing new analgesic therapies (Rice et al., 2008; Mogil, 2009). Using an animal model in which the disease is naturally occurring and similar to the human condition may be more predictive of human clinical efficacy (Lascelles & Flecknell, 2010; Klinck et al., 2017; Lascelles et al., 2019). Feline oral SCC (FOSCC) is one of the most common cancers in cats. While FOSCC is not reported to be virally-induced, its biological activity is quite similar to human papilloma virus-negative head and neck SCC (HPV negative HNSCC) in that both are locally aggressive cancers for which: (1) therapeutic resistance is common, and (2) local tumor progression tends to be a common cause for early disease-specific morbidity and mortality (Bilgic et al., 2015).

While the discomfort caused by FOSCC has not been well-studied, veterinary oncologists anecdotally and widely recognize this disease as being a source of considerable pain, which appears to be clinically similar to that pain which is experienced by people with HNC. Thus, FOSCC could be a useful translational, naturally occurring model of oral cancer pain. However, to be a useful model, robust outcome measures are needed, and currently, none have been developed or described. To address that gap, the goal of this study was to develop and assess methods to measure FOSCC-associated pain. To ensure the greatest translational value, it is important that the outcome measures developed for FOSCC-associated pain closely mimic those used in human medicine. We therefore evaluated three different types of pain and/or sensitivity assessment methods: client questionnaires, clinician-based pain scales and quantitative sensory testing (QST).

Several methods and instruments have been used in human HNC patients for measuring pain, including QOL questionnaires (Niv & Kreitler, 2001; Infante-Cossio et al., 2009). In veterinary patients, pet owner-reported questionnaires have been used to measure QOL and pain-associated behaviors in canine cancer patients (Yazbek & Fantoni, 2005; Lynch et al., 2011; Giuffrida, Farrar & Brown, 2017; Reid, Nolan & Scott, 2018). However, none of these tools were developed specifically for orofacial cancers, or cats. In this study, an owner-reported QOL questionnaire, called the Canine Owner-Reported QOL Questionnaire (CORQ), originally designed to assess QOL in dogs with cancer was used as a guide to construct a tool for measuring QOL in relation to FOSCC, and termed the FORQ—Feline Oral Cancer Owner-Reported QOL Questionnaire (Giuffrida et al., 2018); for ease of reading, it is hereafter referred to (in-text) as the FORQ/CLIENT, to provide readers with a quick reminder that this is the client (pet owner) questionnaire.

Attending physician assessments have been used in human pain medicine to complement patient and caregiver reports; a similar approach can be used in cats, with assessments performed by attending veterinarians (Hadjistavropoulos et al., 1997; Zwakhalen et al., 2004; Kunz et al., 2007). Clinician-completed pain scales have been developed for acute pain in cats (Brondani, Luna & Padovani, 2011; Brondani et al., 2013; Calvo et al., 2014; Reid et al., 2017); instruments have also been developed for evaluating chronic osteoarthritis pain in cats (Lascelles et al., 2007; Benito et al., 2013a; Benito et al., 2013b; Gruen et al., 2015; Klinck et al., 2018b; Klinck et al., 2018a) but none have been developed for the assessment of long-standing orofacial cancer pain. Recently, Stathopoulou et al. (2018) used a modified multifactorial composite pain scale (MCPS) from the Universidade Estadual Paulista Campus de Botucatu (UNESP-Botucatu) to identify the analgesic effect of buprenorphine in cats with gingivostomatitis. Based on their positive results, we felt there was a reasonable chance that a similarly modified scale might also provide a robust measure of feline oral cancer pain; thus, additional modifications to the modified UNESP-Botucatu MCPS scale were applied and studied here; in this manuscript, it is referred to hereafter as the UFEPS/VET to reflect the origin of the scale (see literature citation, where the UNESP-Botucatu MCPS is referred to as the UFEPS) and to quickly orient readers to the fact that this is the questionnaire being administered by the veterinary team (*vs.* the FORQ/CLIENT, which is the pet owner administered tool) (Belli et al., 2021).

In addition to use of clinical metrology instruments such as the UFEPS, complementary information can be gathered through QST. This is potentially useful because pain affects multiple dimensions, and not necessarily all equally (Lascelles et al., 2019). Mechanical and thermal QST represent methodologies that have been used to measure local and distant sensitivity that occurs in humans, and in rodent models (Matos et al., 2011; Zaslansky & Yarnitsky, 1998; Ye et al., 2017). Such methodologies have also been used in dogs with cancer (Monteiro et al., 2018). Electronic von Frey (EVF) and Cochet-Bonnet (COBO) aesthesiometers are examples of mechanical QST (mQST) instruments that have been used for testing cutaneous and/or joint pressure sensitivity, and corneal sensitivity in both dogs and cats (Chan-Ling, 1989; Barrett, Scagliotti & Merideth, 1991; Blocker & Van Der Woerdt, 2001; Briley et al., 2014; Machin, Kato & Adami, 2019). To date, these methods have not been used to assess pain/sensitivity in cats with oral cancer.

A combination of all of these tools formed the basis for the present pilot study, which was performed to begin: (1) validating tools for measuring FOSCC associated pain, and (2) defining various features of oral cancer pain in cats. We hypothesized that it is possible to discriminate between states of normal orofacial sensory processing in healthy cats and abnormal sensory processing in cats with oral cancer by using an owner (client)-reported QOL questionnaire (FORQ/CLIENT), clinician pain scoring instrument (UFEPS/VET), and two forms of mQST.

## Materials & Methods

### Animals

A prospective, non-randomized pilot study was conducted. The study protocol and all procedures were approved by the Institutional Animal Care and Use Committee of North Carolina State University (protocol #18-079-O and #18-082-O) and all pet owners provided oral and written informed consent for all procedures. Enrollment was voluntary. While several of the tumor-bearing cats did subsequently enroll in a fully funded therapeutic trial, no financial incentive (or compensation of any type) was provided for enrollment in this study (Lai et al., 2021). Owned pet cats aged >1 year and weighing >2.5 kg were eligible for this study. Fractious cats were excluded during screening. Cats who were noncompliant (*i.e.,* those for whom assessments could not be completed due to temperament or otherwise) were also excluded. Cats were enrolled into two experimental cohorts: (1) tumor-bearing cats; and (2) healthy controls. In the group of cancer-bearing animals, only cats with a pathology-confirmed diagnosis of sublingual SCC were included (either cytology or histopathology). Enrolled cats were required to discontinue systemically administered anti-inflammatory drugs for at least 72 h and opioids for 48 h prior to enrollment. Complete blood count, serum biochemical analysis, and urinalysis were performed in each cat with oral cancer. For the purposes of clinical cancer staging in cats with SCC, tumor size was measured and if the mandibular lymph nodes were asymmetrical or enlarged, they were aspirated with a fine needle (22 g) for cytologic evaluation. Tumor stage was defined by using an established WHO staging scheme (Owen, 1980). To be enrolled as a healthy control subject, a complete medical history was obtained and physical examination was performed by a veterinarian to ensure the cat was outwardly healthy and free of clinically overt oral disease; neither sedated oral examination nor dental radiography was required. Because cats with sublingual SCC in the present study were >8 years old, and considering that geriatric cats may have subclinical comorbid diseases that can cause pain and/or sensitization (*e.g.*, osteoarthritis) (Lascelles et al., 2007; Gruen et al., 2014), a subset of age-matched healthy control cats were included. In these age-matched controls, lab work (serum biochemistry, hemogram, urinalysis, and thyroid panel) from within the past calendar year was used to supplement physical exam data and ensure “healthy” status. Additionally, while a clear source of potential bias, no attempt was made to mask/blind study personnel to experimental group. Any attempt to do so would have been futile because most cats with oral SCC have clinical signs (*e.g.*, ptyalism, poor body condition score, incomplete grooming) which are readily evident even during distant observation; while these signs are not specific to a diagnosis of FOSCC, they do readily enable distinction between tumor-bearing cats and healthy controls.

### Outcome measures

#### Owner-Reported Quality of Life questionnaire for cats with oral SCC

A recently published scale for evaluating QOL in dogs with cancer (CORQ) was used to help design the preliminary FORQ (preFORQ/CLIENT) –Feline oral cancer Owner- Reported quality of life Questionnaire (see Table S1 and Article S1) (Giuffrida, Farrar & Brown, 2017; Giuffrida et al., 2018). No assumptions were made about validity associated with the CORQ (for dogs) carrying over to FORQ/CLIENT (which was being applied to cats). The preFORQ/CLIENT included a total of 24 questions grouped within four categories: behavior, activity, interaction, and oral/facial discomfort. The initial questions included, and the design, was based on the authors’ experience and discussions, and was considered to have sufficient face validity to move forward (with face validity indicating that the instrument “looks” appropriate for measurement of quality of life, and face validity indicating that the test is designed by experts in the field who believe all relevant face domains are considered) (Brown et al., 2007). Pet owners were requested to score each question with regard to frequency and severity; responses were based on behaviors observed over the preceding 7 days. If a behavior had been seen, the frequency was categorized as: none, rarely (1–2 days), sometimes (3–4 days), usually (5–6 days), or always (every day). Severity was scored as: none, mild, moderate, severe, or very severe. The research team later assigned response numbers, ranging from 0 to 4 with zero reflecting a response of “none” and 4 being either “always” or “very severe”. A total score was calculated; higher scores reflected lower QOL. A 100 mm VAS item was also included at the end of the questionnaire to serve as a single measure of overall QOL; respondents were asked to mark along a line representing somewhere between the “worst imaginable quality of life” (0 mm) and “perfect quality of life” (100 mm). Therefore, on the contrary to FORQ/CLIENT, lower VAS represents lower QOL.

To refine the instrument, each individual item in the preFORQ/CLIENT was analyzed by the non-parametric Mann–Whitney test to evaluate whether that item discriminated between the two groups. Any question with Cohen’s d value that is less than 0.2 or *r* <  0.3 in Spearman’s rank correlation coefficient for both frequency and severity are considered to have small size effect and less relevant to the total preFORQ/CLIENT score (Cohen, 1992; Chan, 2003; Streiner & Norman, 2008); those questions were removed from the questionnaire when creating the “proposed FORQ/CLIENT” (Table 1).

**Table 1:** The proposed FORQ. The proposed FORQ (feline oral cancer owner-reported quality of life questionnaire) represents a refined version of our pre-FORQ; low-performing items have been removed to improve efficiency of the instrument, and this is the final version being put forward for future and continued validation.

During the past 7 days, my cat:	No	If YES, *how often* did your cat have it?				If YES, *how severe* was it usually?			
		Rarely	Sometimes	Usually	Always	Mild	Moderate	Severe	Very severe
**Behavior**									
• Had low energy?	$○ $	$○ $	$○ $	$○ $	$○ $	$○ $	$○ $	$○ $	$○ $
• Was reluctant to wake up?	$○ $	$○ $	$○ $	$○ $	$○ $	$○ $	$○ $	$○ $	$○ $
• Had altered mood?	$○ $	$○ $	$○ $	$○ $	$○ $	$○ $	$○ $	$○ $	$○ $
• Had trouble getting comfortable?	$○ $	$○ $	$○ $	$○ $	$○ $	$○ $	$○ $	$○ $	$○ $
• Growled or groaned when resting?	$○ $	$○ $	$○ $	$○ $	$○ $	$○ $	$○ $	$○ $	$○ $
• Could not maintain hygiene (i.e., grooming)?	$○ $	$○ $	$○ $	$○ $	$○ $	$○ $	$○ $	$○ $	$○ $
• Had decreased appetite?	$○ $	$○ $	$○ $	$○ $	$○ $	$○ $	$○ $	$○ $	$○ $
• Drank less water than usual?	$○ $	$○ $	$○ $	$○ $	$○ $	$○ $	$○ $	$○ $	$○ $
**Activity**									
• Had trouble with mobility?	$○ $	$○ $	$○ $	$○ $	$○ $	$○ $	$○ $	$○ $	$○ $
• Did not do what he/she likes (e.g., chasing, playing, etc.)?	$○ $	$○ $	$○ $	$○ $	$○ $	$○ $	$○ $	$○ $	$○ $
• Did not act like his/her normal self?	$○ $	$○ $	$○ $	$○ $	$○ $	$○ $	$○ $	$○ $	$○ $
• Had decreased enjoyment of life?	$○ $	$○ $	$○ $	$○ $	$○ $	$○ $	$○ $	$○ $	$○ $
• Did not sleep well?	$○ $	$○ $	$○ $	$○ $	$○ $	$○ $	$○ $	$○ $	$○ $
**Interaction**									
• Showed a decreased amount of affection?	$○ $	$○ $	$○ $	$○ $	$○ $	$○ $	$○ $	$○ $	$○ $
**Oral/facial discomfort**									
• Had excessive drooling?	$○ $	$○ $	$○ $	$○ $	$○ $	$○ $	$○ $	$○ $	$○ $
• Had difficulty eating his/her normal food?	$○ $	$○ $	$○ $	$○ $	$○ $	$○ $	$○ $	$○ $	$○ $
• Was offered and had trouble eating soft food?	$○ $	$○ $	$○ $	$○ $	$○ $	$○ $	$○ $	$○ $	$○ $
• Had trouble lying down his/her head?	$○ $	$○ $	$○ $	$○ $	$○ $	$○ $	$○ $	$○ $	$○ $
• Felt discomfort or pain near the mouth?	$○ $	$○ $	$○ $	$○ $	$○ $	$○ $	$○ $	$○ $	$○ $

**Notes.**

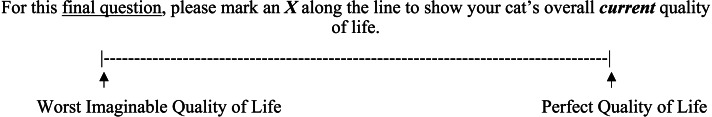

#### Clinician pain scoring instrument for assessing orofacial pain in cats with oral SCC

The UNESP-Botucatu multidimensional composite pain scale for cats with oral diseases was modified to better reflect common ailments and dysfunction that is observed in cats with oral cancer; as mentioned above, the modified scale is referred to here as UFEPS/VET (see Table S2; changes made here are indicated in bold; arterial blood pressure was omitted because of our concerns that its measurement may not be reliable, and can cause undue stress) (Brondani et al., 2013; Stathopoulou et al., 2018). No assumptions were made about validity associated with the UNESP-Botucatu pain scale carrying over to the UFEPS/VET. In the category of miscellaneous behavior, the abnormal behavior of “lick and/or bites the surgical wound” was changed to “licks, has ptyalism and/or chattering (jaw shakes)”. Categories in the instrument pertained to: miscellaneous behavior; reaction to palpation around the mouth and on the head; vocalization; posture; comfort; activity; attitude; or appetite. Each category was scored on a 4-point scale, with 0 indicating normal (or no change) and 3 denoting significantly altered behavior. The clinician gave a score based on the description provided. In the evaluation of appetite, a small amount of canned commercial cat food was offered. In instances where fasting had to be performed at the time of the pain scoring (*e.g.*, in preparation for general anesthesia), the scores were based on the amount of food the cat had consumed at its most recent meal.

#### Electronic von Frey

An EVF apparatus (BIO-EVF3, Bioseb, Chavillecedez, France) was used to assess mechanical sensory thresholds. A plastic pipette tip was loaded on the mounting accessory of a hand-held device that can record the applied force. The force was gradually increased manually until a positive response was elicited, with the upper cut-off limit set as 500 g. Positive responses included vocalization, head withdrawal, paw withdrawal, attempt to ‘bat’ or paw at the device, or trying to bite the device. Tests were repeated five times with an interstimulus interval of approximately 1 min. The mean of all five trials was used for data analysis. Lower thresholds indicate greater sensitivity. Measurements were acquired at anatomic sites based on anatomy of the three branches of the trigeminal nerve, which mediates somatosensory input from most of the orofacial region. The mandible and tongue are innervated by the mandibular branches, and the maxillary cutaneous region is innervated by maxillary branches of the trigeminal nerve. Since the input from one area of the face may result in changes in sensitivity in another facial region, we selected four measurement sites (Fig. S1): (1 and 2) just medial to the bilateral mandibulae (intermandibular space), (3) the ipsilateral maxilla, along the path of the maxillary branch, and (4) on the dorsal aspect of the right metacarpus –a site distant to the mouth, which served as the control (Jääskeläinen, 2004). For subjects in the healthy cat cohort, the right maxilla was always tested; other measurement sites (left and right intermandibular space and right metacarpus) were the same as in the test group.

#### Cochet-Bonnet aesthesiometer

The afferent impulses of corneal reflexes are mediated by the ophthalmic division of the trigeminal nerve. Corneal touch threshold (CTT) values were measured using a Luneau Cochet-Bonnet aesthesiometer (Western Ophthalmics, Lynwood, WA) with a monofilament nylon fiber of 0.12 mm diameter, as previously described (Blocker & Van Der Woerdt, 2001). Testing was performed on all enrolled cats, unless they had pre-existing corneal diseases (*e.g.*, corneal scarring, ulcers). The cats were gently manually restrained with their head up; their body was cradled between the restrainer’s chest and forearm. All measurements were made by a single veterinarian, in a quiet and well-lit room. The instrument was held perpendicular to the cornea, and the aesthesiometer filament was applied centrally, on the left cornea. Testing started with a filament length of 60 mm. The cornea was touched with that length filament, and then if there was no blink response, the filament was shortened by five mm to increase resistance. This process was repeated until a corneal blink was elicited. Once the animal blinked, that same length filament was re-applied a total of 3 times. If that length elicited blink on at least 2 of 3 applications, that length was defined as the CTT. If that length only elicited blink for 1 of 3 applications, progressive shortening continued until a length was identified that did elicit blink in 2 of 3 applications (*i.e.,* 1 of 3 was defined as a false positive, and 2 or 3 of 3 was a true positive). The absence of corneal damage was confirmed *via* fluorescein staining at the conclusion of each testing session.

For both mQST assays, cats were fully conscious (no sedation) and gently restrained by a single trained technician. No animal was scruffed or forcibly restrained for this testing. Testing was performed in a dedicated room that was quiet, and had no animals in it other than cats; the room is in a veterinary hospital and it is possible that the scent and/or noise of dogs may have been present during some testing sessions, but all reasonable efforts were made to minimize such potential environmental influences.

### Experimental design

For each outcome measure, we assessed: (1) reliability; (2) discriminatory ability; and (3) responsiveness.

To test reliability (test –retest reliability / intra-rater reliability), Experiment 1 included two measurements on the same subject in both cancer-bearing cats and healthy cats with the assessment being performed by the same person at each timepoint. FORQ/CLIENT did not undergo reliability testing. Measurements of UFEPS/VET, EVF, and COBO aesthesiometer were repeated with at least 6 h between the two tests in the control cats (to facilitate a single outpatient visit) and at least 12 h between the two tests in the cancer cats (overnight, since the cats were to be hospitalized for an unrelated therapeutic intervention trial to begin shortly after the 12 h retest). Tests were performed in the same environment on each subject by the same researcher (YHL) each time. No attempt was made to assess inter-rater reliability within this experiment.

In Experiment 2, cats with oral cancers were compared to healthy cats without oral disease to examine the discriminative validity of each assessment. We performed the FORQ/CLIENT (including VAS), UFEPS/VET, EVF, and COBO aesthesiometer on cats with and without sublingual SCC. Where applicable, initial test (not retest) data from Experiment 1 were used for analysis in Experiment 2. As noted above, a single unmasked observer made all assessments.

Experiment 3 investigated the responsiveness validity of the assessments using cats with sublingual SCC. Assessments were conducted three times (at baseline, 30 min after buprenorphine, and the next day). The buprenorphine (0.3 mg/mL injectable solution, Par Pharmaceutical, Chestnut Ridge, NY) was administered buccally as a liquid, once, with a dose of 0.02 mg/kg as described previously (Robertson et al., 2005).

### Statistics

Normality of the data was evaluated by the Shapiro–Wilk test. Test –retest reliability (Experiment 1) evaluation was performed using intra-class correlation coefficient (ICC) and Bland-Altman analysis. In Experiment 2, comparisons of preFORQ/CLIENT and UFEPS/VET between healthy control and cancer cats were performed using the non-parametric Mann–Whitney U-test. Unpaired student *t*-tests were used to compare the results of EVF and COBO aesthesiometer between two groups. The effect sizes of preFORQ/CLIENT and UFEPS/VET were evaluated by Cohen’s d calculation. Based on the value of Cohen’s d, effect size can be considered low (0.2), medium (0.5), or large (0.8). Item-total correlation was evaluated by Spearman’s rank correlation coefficient. Coefficients with value of *r* <  0.3 were considered poor, 0.3 <*r* ≤ 0.5 were fair, 0.5 <*r* ≤ 0.8 were moderately strong, and >0.8 were very strong (Chan, 2003). Questions were removed if the Cohen’s *d* <  0.2 or the correlation coefficient *r* <  0.3 (Cohen, 1992; Streiner & Norman, 2008). The reliability of the questions in preFORQ/CLIENT and UFEPS/VET was evaluated by Cronbach’s *α* analysis (Cronbach, 1951; Taber, 2018). The interpretations of Cronbach’s *α* were as follows: <0.35, low reliability; 0.35 to 0.7, medium reliability; >0.7, high reliability. Receiver operating characteristic (ROC) curves were built based on total scores for preFORQ/CLIENT (including VAS), EVF, and COBO aesthesiometer to determine the cut-off point. The cut-off point was identified by calculating Youden index (YI), which represents the greatest point of specificity and sensitivity (Streiner & Cairney, 2007). Area under the curve (AUC) ≥ 0.9 indicates outstanding capacity of discrimination, 0.8 to 0.9 is considered excellent, 0.7 to 0.8 is acceptable, and ≤ 0.5 suggests no discrimination (Mandrekar, 2010). In Experiment 3, either the Wilcoxon matched-paired test, or paired *t*-test, was performed on data collected before buprenorphine and 30 min after buprenorphine. Post-hoc power analyses were performed by using G*Power 3.1.9.7 (Heinrich-Heine-Universität Dusseldorf, Germany). The level of significance was defined as *P* < 0.05. All statistical analyses were performed using commercial software (Prism 7; GraphPad Software, San Diego, CA).

## Results

### Animals

All healthy control cats were client-owned pet cats specifically recruited for this study. The cats with sublingual SCC (hereafter described as “cancer cats”) were referred to our Radiation Oncology service, from veterinary practices in North Carolina ( *n* = 6) and Massachusetts (*n* = 1) between September 2018 and November 2019. Each of these cancer cats was classified as having WHO stage III OSCC (no evidence of lymph node involvement or distant metastasis) at the time of enrollment. A total of 18 healthy cats and seven cancer cats were initially included in the study. However, one cancer cat, and two healthy cats were noncompliant for the EVF assessment; complete orofacial region assessment could not be performed in those two healthy cats, and right forelimb thresholds could not be obtained in the cancer cat. Thus, complete pain/sensitivity evaluations were feasible in 16/18 (89%) healthy cats and 6/7 (86%) cancer cats. The noncompliant animals were excluded from the study, and their data was not included in any analysis (*i.e.,* data analyses reflect data from 16 healthy control cats and 6 cancer cats). Healthy control cats were assessed as a whole, group, and a subset analysis was performed for cats aged >8 years (6 out of 18 healthy controls; see *Age-matched comparison* below). Demographics of the cats whose data were included in the analyses are summarized in Table S3 .

### Experiment 1: Test –retest reliability

**Figure 1:**
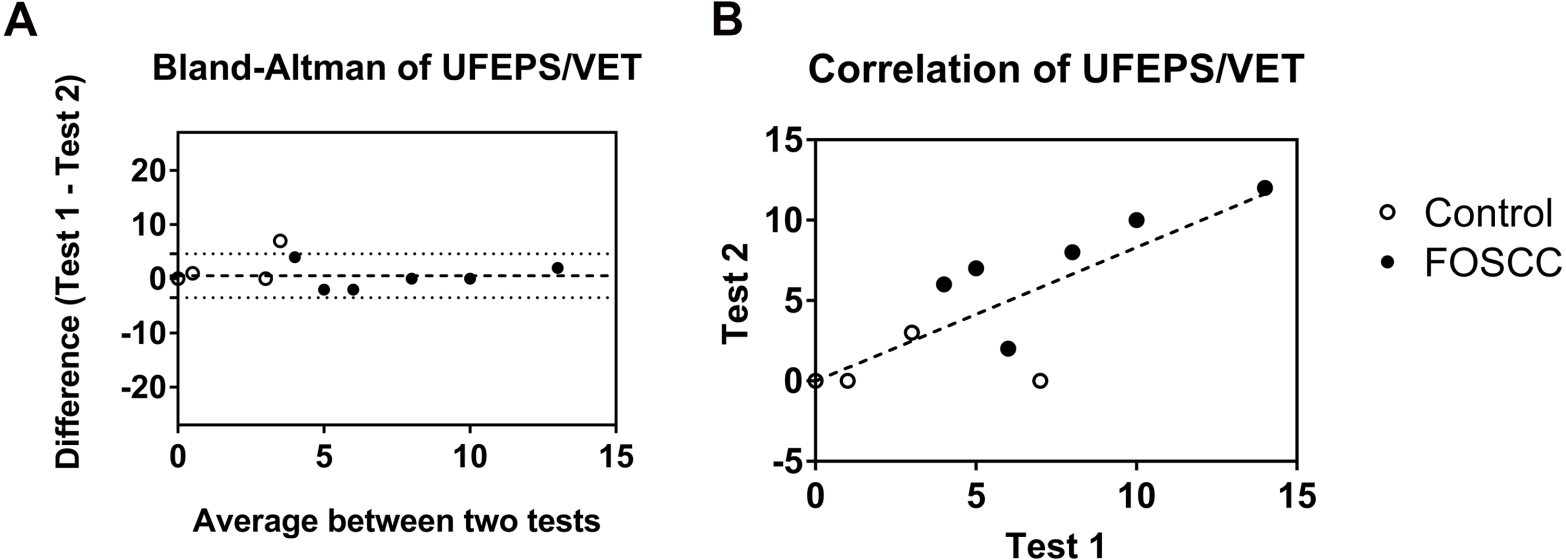
Test –retest reliability evaluation of clinician-based feline orofacial cancer pain scoring (UFEPS/VET). (A) The difference between two replicates of the UFEPS/VET are plotted against the mean of both repeats. The dashed line represents a bias of 0.5556; the dotted lines represent the 95% limits of agreement (LoA ), 4.602 and −3.491. (B) The two scores had good and significant correlation (*r*^2^ = 0.77, *P* < 0.0001). Each dot represents an individual cat; open dots denote healthy control cats; closed/solid dots denote cats with sublingual SCC.

There were 12 healthy cats and five cancer cats enrolled in Experiment 1 (cancer cat #6 was unable to contribute retest data due to logistical challenges with data collection, not due to subject noncompliance). The test –retest reliability was good for each pain assessment instrument (UFEPS/VET, EVF, and COBO). The UFEPS/VET used in the study can be found as Table S2. The ICC was 0.919 (95% CI [0.776–0.971]). A Bland-Altman plot (Fig. 1A) shows the difference between test and retest on the *Y*-axis and the mean of two tests on the *X*-axis; bias between test and retest of UFEPS/VET was 0.5556, which indicated a slightly higher score on the first test. Data from the two tests were positively correlated (r^2^ = 0.77, *P* < 0.0001; Fig. 1B).

For pressure algometry, the ICC for the maxillary measurement site was 0.73 (95% CI [0.24–0.91]) for average measures. At the metacarpal measurement site, the ICC was 0.68 (95% CI [0.10–0.88]). At the intermandibular sites, these values were 0.73 (95% CI [0.40–0.90]) for the left side, and 0.72 (95% CI [0.37–0.89]) for the right side. The Bland-Altman plot showed most of the differences between the two tests lay between the 95% limits of agreement (LoA) (Fig. S2, left panel). The biases were 5.5, 27.9, 35.3, and 13.6 g in the EVF tests on the right and left intermandibular space, right maxilla, and right metacarpus, respectively. The variability was greater at higher thresholds. The correlations between test and retest were statistically significant at all four testing sites (*P* = 0.0006 –0.0365, Fig. S2, right panel), which indicated good test –retest reliability for EVF.

The ICC for COBO measurements was 0.21 (95% CI [−0.29–0.62]) for single measures and 0.34 (95% CI [−0.81–0.76]) for average measures. Bland-Altman plots of COBO measurements showed the bias between two tests was 0 cm and the difference between two tests were within LoA (*i.e.,* between −1.9 and +1.9) (Fig. S3). The correlation between the two tests was not statistically significant (*P* = 0.1823), but the CTTs in cats with sublingual cancers were generally higher than control cats.

Since our test –retest experiments have shown good reliability, we used the first measurement for statistical analysis in Experiment 2: discriminatory validity (except for FORQ/CLIENT, which was answered once by the cat owners in the study).

### Experiment 2: Discriminatory validity

#### Preliminary feline oral cancer owner-reported quality of life questionnaire (preFORQ/CLIENT)

**Figure 2:**
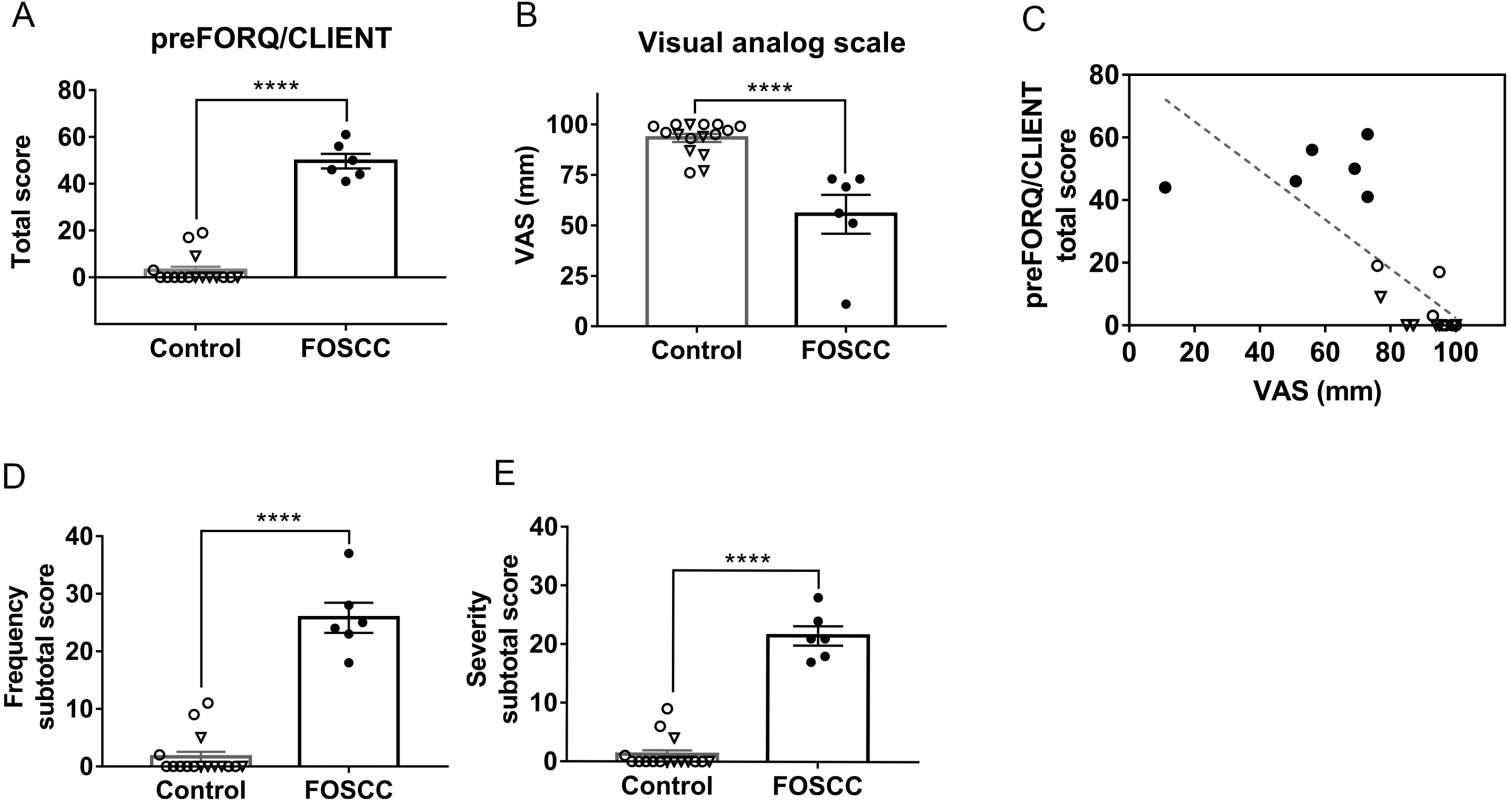
Preliminary feline oral cancer owner-reported QOL questionnaire (preFORQ/CLIENT) results. Worse QOL in cats with oral cancer pain is evidenced by both the: (A) significantly higher total scores; and (B) significantly lower VAS in cancer cats. (C) The correlation between preFORQ/CLIENT total scores and VAS was statistically significant (*P* < 0.0001, r = −0.77, *r*^2^ = 0.6). (D) The frequency and (E) severity scores were significantly higher in cats with oral cancer pain, ^∗∗∗∗^*P* < 0.0001, Mann–Whitney test. Each dot represents the score of individual cats. Values from healthy cats are denoted by open dots; triangular shaped dots denote aged healthy control; closed/solid dots represent cats with sublingual SCC. All error bars depict SEM.

There were 16 healthy cats and six cancer cats from whom data were included in Experiment 2. The discriminatory analysis is summarized in Table 2. The mean ± SD of preFORQ/CLIENT total score in cancer cats was significantly higher (49.67 ± 7.6) than healthy cats (3 ± 6.3, *P* < 0.0001, Fig. 2A). The VAS was significantly lower in cats with sublingual SCC than healthy controls (*P* < 0.0001, Fig. 2B); remember: the lower VAS suggests lower QOL, which is opposite of how the FORQ/CLIENT scores change relative to QOL. The mean ± SD of VAS in cancer cats was 55.5 ± 23.65 mm, compared to 93.3 ± 7.9 mm in healthy cats. At the cut-off value of <74.5 mm, the sensitivity and specificity of VAS were both 100%, with the YI of 1. As would be expected, the total scores for preFORQ/CLIENT and VAS from all respondents were inversely proportional (*r* =  −0.77, r^2^ = 0.6, *P* <  0.0001, Fig. 2C). The mean ± SD of subtotal scores in terms of frequency and severity were both significantly higher in cancer patients (25.83 ± 6.37 and 21.5 ± 4.04, *P* < 0.0001, respectively, Figs. 2D and 2E) than healthy cats (mean = 1.68 ± 3.52 and 1.25 ± 2.7, respectively). Apart from the category of “interaction”, the total scores in all other categories (*i.e.,* general behavior, activity, and orofacial discomfort) were significantly higher in cancer cats than in healthy controls (*P* < 0.0001, respectively, Table S4). The Cronbach’s *α* value of preFORQ/CLIENT was 0.91, which suggests strong reliability (Cronbach, 1951).

**Table 2:** Discriminatory analysis of outcome measures.

**Outcome measures**	**Intraclass correlation coefficient**	**Sensitivity (%)**	**Specificity (%)**	**Youden index**	**Area under curve (AUC) ± SEM**	**Effect size**	**Post-hoc power**
FORQ/CLIENT	Not available	100	100	1	1 ± 0	6.69	1
VAS	Not available	100	100	1	1 ± 0	2.14	1
UFEPS/VET	0.92	100	94	0.94	0.97 ± 0.04	2.44	1
EVF-right intermandibular space	0.72	100	94	0.94	0.97 ± 0.04	2.8	1
EVF-left intermandibular space	0.73	100	94	0.94	0.99 ± 0.02	2.59	1
EVF-right maxilla	0.73	100	56	0.56	0.8 ± 0.10	1.26	0.81
EVF- right metacarpus	0.68	83	94	0.77	0.95 ± 0.05	2.5	1
COBO aesthesiometer- left cornea	0.21	100	75	0.75	0.92 ± 0.06	2.08	1

Each question item was assigned a number for further analysis (Table S1). By reviewing the heat plot, the most frequent observations in cancer cats were excessive drooling and trouble eating (QF-19 and -20, Fig. S4A); in regards to severity, the mean score in drooling were the highest in cancer cats when compared to other questions (QS-19, Fig. S4B). Analysis of individual questions showed that 11 of 24 questions were able to discriminate between cancer and healthy cats (Table S5 ). Using a cut-off point of <27.5 for the sum of frequency and severity scores, both the sensitivity and specificity of preFORQ/CLIENT were 100% and the YI was 1.

To refine our questionnaire, and define a FORQ/CLIENT that can be used in future research, we excluded those questions with Cohen’s d value < 0.2 or *r* <  0.3 in Spearman rank correlation coefficient for both frequency and severity. The refined, proposed FORQ/CLIENT is a 19-item questionnaire (Table 1). The removed questions were: had trouble positioning to defecate/urinate (Q-09), lost balance (Q-11), unwilling to be accompanied (Q-16), did not like to be pet (Q-18), and defensive behavior when touching the head (Q-24). The refined FORQ/CLIENT has an improved Cronbach *α* value (0.92) and the sensitivity and specificity remained 100% when using a cut-off point of <25.5.

#### Feline orofacial pain scale for cancers (UFEPS/VET)

**Figure 3:**
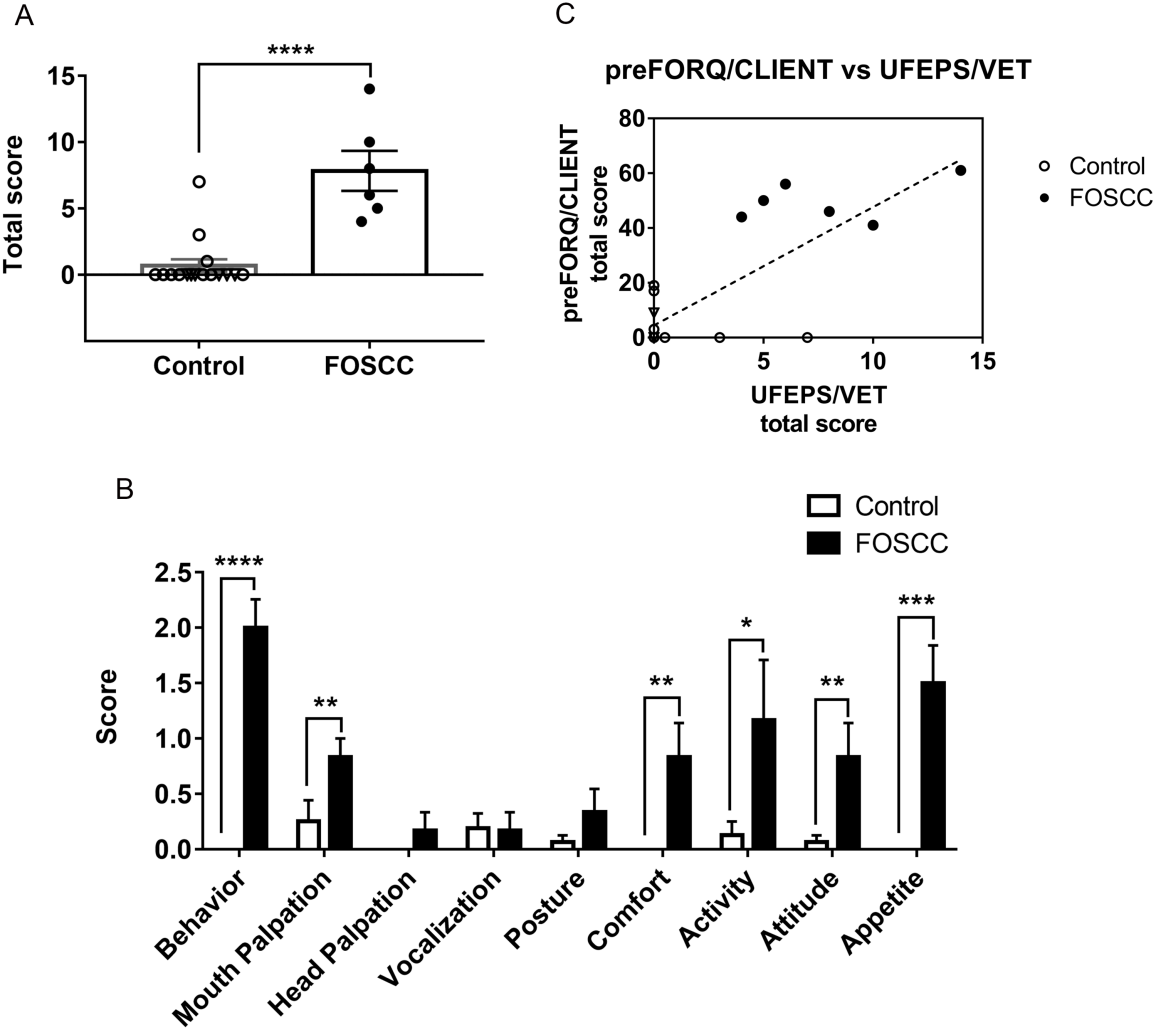
Clinician-based feline orofacial cancer pain scoring (UFEPS/VET). (A) Cats with oral SCC had a significantly higher total score compared to healthy cats. (B) Specifically, cats with oral tumors had significantly higher scores in the UFEPS/VET categories of miscellaneous behavior, mouth palpation, comfort, activity, attitude and appetite. (C) The UFEPS/VET scores were significantly correlated to preFORQ/CLIENT scores (*P* = 0.0002, *r*^2^ = 0.76). ^∗^*P* < 0.05, ^∗∗^*P* < 0.01, ^∗∗∗^*P* < 0.001, ^∗∗∗∗^*P* < 0.0001, Mann–Whitney test. Each dot represents the score of individual cats. Values from healthy cats are denoted by open dots; triangular shaped dots denote aged healthy control; closed/solid dots represent cats with sublingual SCC. All error bars depict SEM.

Compared to healthy cats, cancer cats had significantly higher total scores in UFEPS/VET, indicating that cats with sublingual SCC exhibit more pain-related behaviors (Fig. 3A). Cancer cats had significantly higher scores in miscellaneous behavior (*P* < 0.0001), mouth palpation (*P* = 0.0043), comfort (*P* = 0.0021), activity (*P* = 0.0349), attitude (*P* = 0.0075), and appetite (*P* = 0.0002), as shown in (Fig. 3B). However, head palpation, vocalization, and posture did not show significant differences between healthy cats and cats with sublingual SCC. Cronbach’s *α* was 0.84, indicating high reliability. With a cut-off point of >3.5 for total score, the YI was 0.94; at this cut-off, sensitivity and the specificity were 100 and 94%, respectively. The AUC was 0.97 ± 0.04, indicating that UFEPS/VET has high discriminatory capacity. The total UFEPS/VET score was positively correlated with the preFORQ/CLIENT score (*r*^2^ = 0.58, *P* = 0.0002, Fig. 3C) and the proposed, refined FORQ/CLIENT score (*r*^2^ = 0.72, *P* <0.0001).

In the miscellaneous behavioral category, the majority of observations in cancer cats were: ‘laying down and quiet’ (n = 5/6), and ‘licks, ptyalism, or chattering’ (n = 4/6) (see Table S2). In attitude evaluation, five out of six cats with sublingual SCC were uninterested in interacting with the observer and one cat showed both disinterest in the observer and indifference to its surroundings. Cats with sublingual SCC tended to show interest in food, but did not continue consumption after a few bites.

Cohen’s d values for all items in UFEPS/VET were large (>0.8), except for “vocalization” (d = −0.04). Spearman rank correlation coefficient test showed “head palpation” had low correlation to the total score (*r* = 0.25). After removing “vocalization” and “head palpation”, the Cronbach’s *α* = 0.88; at a cut-off point of >3.5, the sensitivity, specificity, and YI remained 100%, 94%, and 0.94, respectively, but the AUC increased to 0.98 ± 0.03.

#### Electronic von Frey and Cochet-Bonnet Aesthesiometer

**Figure 4:**
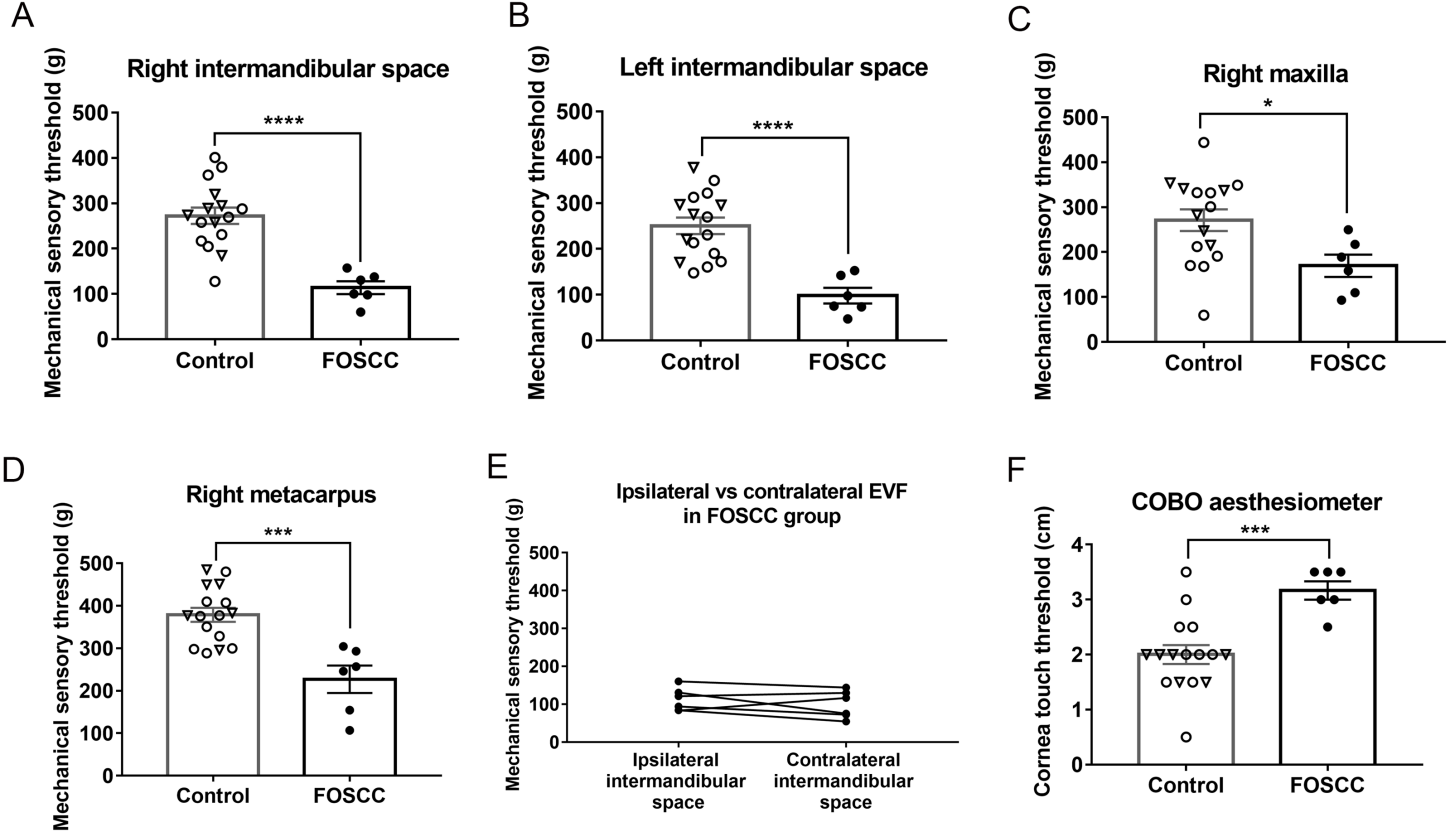
Mechanical quantitative sensory tests (QST). Cats with sublingual SCC were more sensitive to pressure (EVF test) at the (A) right and (B) left intermandibular test sites, (C) maxillary region, and (D) right metacarpus. (E) There was no significant difference between the QST values on the ipsilateral (tumor) side *versus* the opposite (contralateral) side of the mandibular region in cats with sublingual SCC; paired *t*-test. (F) Cats with oral cancer were more sensitive to mechanical stimuli on central corneal touch (CCT) measured with the COBO aesthesiometer. Each dot represents the measurement of individual cats. Values from healthy cats are denoted by open dots; triangular shaped dots denote aged healthy control; closed/solid dots represent cats with sublingual SCC. ^∗^*P* < 0.05, ^∗∗∗^*P* < 0.001, * ^∗∗∗^*P* < 0.0001*,* unpaired *t-* test. All error bars depict SEM.

Cats with sublingual SCC were significantly more sensitive to pressure (*i.e.,* had lower mQST thresholds) at both sides of the intermandibular space (Figs. 4A and 4B, *P* < 0.0001 on both right and left side), and at the right maxillary region (Fig. 4C, *P* = 0.0273). The mean differences of the mechanical sensory threshold between healthy and cancer cats on the right intermandibular space, left intermandibular space, and right maxillary region were 158.5 ± 31.02, 152.2 ± 31.45, and 101.1 ± 42.58 grams, respectively. Based on the ROC curve, the sensitivity can reach 100% at either side of intermandibular space, and the specificity is 94%. At right maxillary region, the sensitivity and specificity were 100 and 56%, respectively. Interestingly, decreased thresholds were observed at the metacarpal site (*P* = 0.0002) in cancer cats compared to healthy controls (227 ± 32.32 *versus* 378.7 ± 16.43 g, Fig. 4D). The sensitivity and specificity of EVF on metacarpal region were 83.33 and 93.75%, respectively. The AUC ranged from 0.8 to 0.95 at all EVF test sites, which indicates excellent discriminatory ability. In cancer cats, five out of six cats had tumors that originated from the right side and one out of the six cats (FOSCC#4) had a tumor originating from the left side. However, all of the cats had masses that crossed the midline and occupied the base of the tongue. There was no significant difference between the mQST thresholds at ipsilateral and contralateral sides of the intermandibular region in cats with sublingual SCC (Fig. 4E, *P* = 0.883).

Previous work has demonstrated that healthy domestic short-haired cats have similar CTT values in the right and left eyes (Blocker & Van DerWoerdt, 2001). To reduce procedure time and stress on animals, we only performed the COBO aesthesiometer test on left corneas. In contrast to the threshold value measured by EVF, higher CTT values indicate that a subject is more sensitive to corneal touch. CTT values from the left cornea showed that cats with sublingual SCC were more sensitive than control animals (Fig. 4F, *P* = 0.0009); on average, healthy cats were able to sustain higher pressures (1.17 ± 0.3 cm shorter filament). At the cut-off point of >2.25 cm, sensitivity and specificity of COBO for discrimination between cancer cats and healthy cats were 100 and 75%, respectively. Based on the ROC curve, the COBO has outstanding discriminatory capacity (AUC 0.92 ± 0.06).

Sex differences have been shown when measuring orofacial sensitivity using QST in humans (Costa et al., 2019). We compared the threshold values measured by EVF and COBO aesthesiometer between healthy castrated males (*n* = 6) and spayed females (*n* = 10), but did not identify statistically significant differences between sexes (Fig. S5). There were too few cancer-bearing cats to make a statistically relevant comparison.

### Age-matched comparison

In a subset analysis, the outcome measurements from 6 geriatric cats (aged 8-15 years, with median of 10 years) in the healthy control group were compared to the 6 cancer cats. The body weight (mean ± SD) of cancer cats was less (3.7 ± 0.58 kg) than healthy counterparts (4.7 ± 0.56 kg, *P* = 0.0106). Cancer cats had significantly higher scores in terms of preFORQ/CLIENT and UFEPS/VET than aged healthy cats (Fig. S6). Cancer cats also showed significantly decreased sensory thresholds in EVF and COBO aesthesiometer tests (Fig. S6 and Table S6).

### Experiment 3: Responsiveness validity

**Figure 5:**
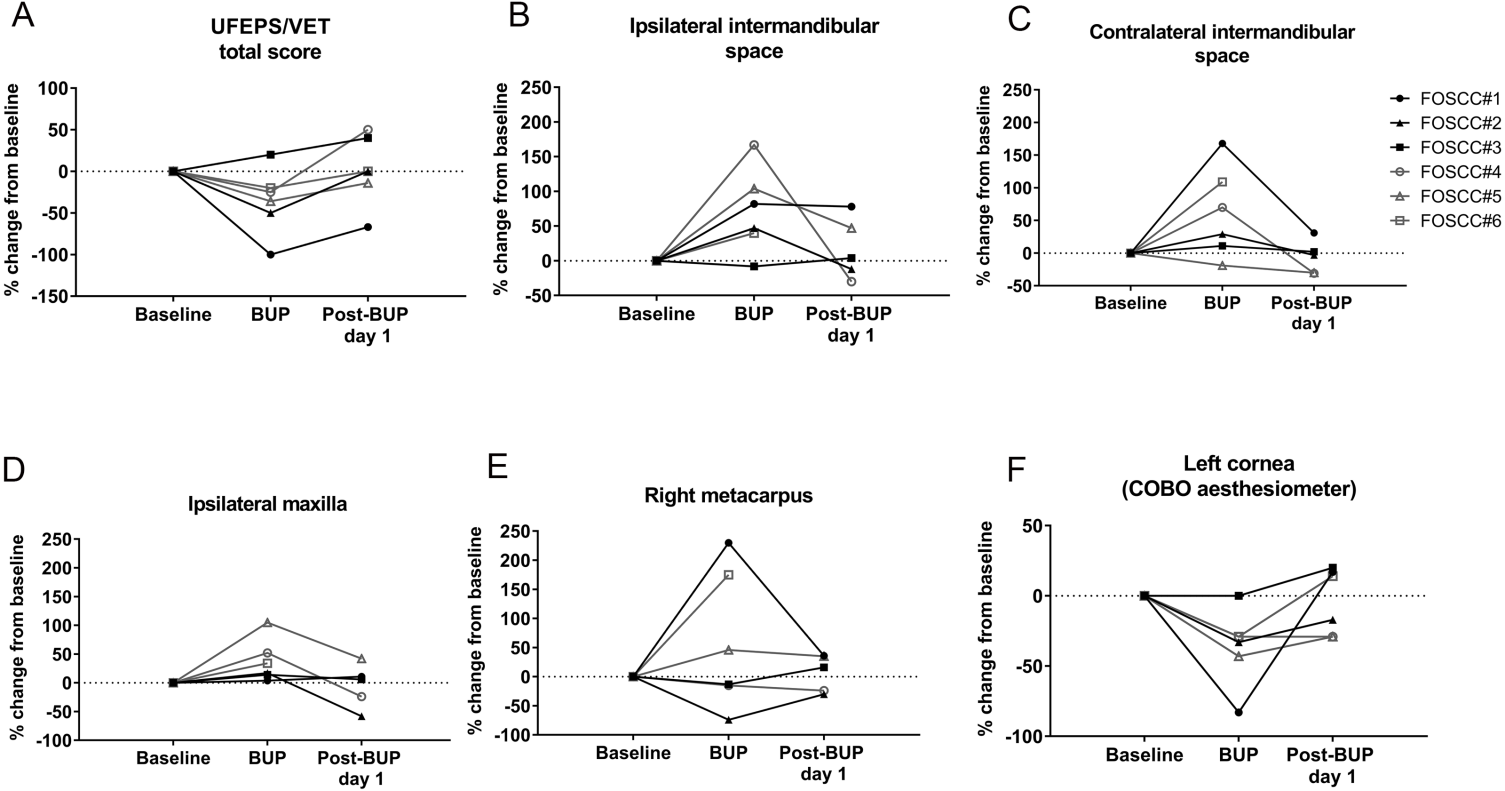
Analgesic responsiveness of each outcome measure in cats with oral cancer. Responsiveness testing of outcome measures in cats with oral cancers via: (A) UFEPS/VET; EVF testing at the (B) ipsilateral and (C) contralateral intermandibular sites, (D) ipsilateral maxilla, and (E) right metacarpus; and (F) COBO aesthesiometer testing. Cats had altered thresholds in orofacial pain measurements after buccal administration of buprenorphine; thresholds returned to baseline by the next morning. Significantly increased mechanical sensory thresholds at the ipsilateral intermandibular sites were observed 30 min after buprenorphine administration, and similarly, the length of COBO filament required to elicit response became shorter (*i.e.,* the pressure threshold increased) with buprenorphine administration (*P* = 0.04 and 0.02, respectively, see Table S7).

UFEPS/VET, EVF, and COBO measurements were performed before and 30 min after administering an analgesic drug (buprenorphine 0.02 mg/kg, buccally) to six cats with SCC of the tongue. The mechanical sensory threshold at the ipsilateral intermandibular site, and CTT values were significantly improved after buprenorphine administration (Fig. 5, the details can be found in Table S7). The mechanical sensory threshold increased by 66.85 ± 24.48 g (*P* = 0.04) at the ipsilateral intermandibular site after giving buprenorphine, and after buprenorphine, cancer cats could withstand corneal pressures that were higher (1.17 ± 0.33 cm shorter filament; *P* = 0.017). Although not statistically significant, other tests (including UFEPS/VET, and EVF at other sites) also showed a trend toward decreased pain/sensitization after providing buprenorphine; this is notable given the small sample size, where lack of statistical significance may reflect true biology or statistical error. Based on post hoc power analysis, with the exception of COBO testing, power was low (< 0.8) for all comparisons in Experiment 3 (Table S7). Nonetheless, results are biologically plausible, and for each assay, the pain score/sensitivity returned to baseline (*i.e.,* pre-buprenorphine levels) by the next morning.

## Discussion

This study reports on the adaptation and initial testing of various outcome measures that should enable robust research in a naturally occurring feline model of oral cancer pain. Based on our preliminary studies, the methods described herein appear to have good intra-rater reliability within a single observer; furthermore, we provide evidence that these assays effectively discriminate between healthy and tumor-bearing cats, and some are responsive to analgesic therapy. Interestingly, our results also suggest that cats with tongue cancer not only develop pain in their mouth, but also seem to have regional peripheral, and widespread sensitization; this is consistent with the widespread somatosensory sensitization that is reported in human HNC patients and which can be measured in other animal models of oral cancer pain (Ye et al., 2017).

One goal of this study was to develop methods for assessing pain in a naturally occurring feline model of oral cancer. Pain can affect overall QOL, thus in our study, we attempted to measure QOL rather than health-related QOL, which only focuses on the impact of a specific illness on QOL (Reid, Nolan & Scott, 2018). Given that a canine owner-reported QOL questionnaire for cancer patients had been developed and published, we used this to guide the initial construction of an instrument to evaluate the QOL of cats with oral cancers. Through basing this instrument on the canine cancer QOL instrument, and discussions between the authors, we believed the initial FORQ/CLIENT had sufficient face validity to move forward into testing and refinement. Our initial (preliminary) version of the FORQ/CLIENT showed high reliability and good discriminative function; it distinguished healthy cats from those with sublingual SCC. However, some individual questions’ effect size was small, leading us to omit them from the proposed version of the FORQ/CLIENT. In our study, cats with oral cancers did not have significant mood alteration, reluctance to wake up, or vocalization when resting, and they did have normal interactions with the owners, which is different from observations made in canine cancer patients (Giuffrida et al., 2018). This discrepancy may be due to the natural characteristics of different species. We also found that “less drinking” was not a good indicator in cats with sublingual cancers. Although cats with sublingual cancers decreased their normal food intake, it is known that mouth dryness and pain are common in human patients with advanced cancers. We therefore theorize that increased water intake could possibly indirectly indicate mouth dryness and/or pain in these tongue cancer-bearing cats (Oneschuk, Hanson & Bruera, 2000). Overall, similarly negatively altered QOL has been reported in human HNC patients (Gotay & Moore, 1992; Terrell et al., 2004), which supports the potential translational utility of this outcome measure.

To complement these pet owner derived assessments of QOL, we also tested a clinician-based clinical metrology instrument focused on measuring the impact of oral pain. UFEPS/VET scores were significantly greater for oral cancer-bearing cats than control cats, and UFEPS/VET scores were highly correlated to FORQ/CLIENT responses. The UFEPS/VET assesses various behavioral changes that indicate pain and/or discomfort, not only around the oral region, but also in terms of general comfort, activity and attitude. The Cohen’s d value shows small effect size for vocalization, and the Spearman rank correlation coefficient showed head palpation related poorly to the total score; thus future iterations of the UFEPS/VET might exclude these parameters. Most cancer-bearing cats maintained interest in food; however, tumor-associated dysphagia is known to reduce food intake (which is reflected in the “difficulty eating food” data in FORQ/CLIENT); thus, in future iterations of the UFEPS/VET, we may consider revising the appetite scoring to reflect this disconnect between appetite and ability to successfully prehend food. Additionally, the value of UFEPS/VET may be improved through the incorporation of facial expression assessment; both ear position and muzzle shape have been used to in feline acute pain evaluations (Holden et al., 2014; Reid et al., 2017; Evangelista et al., 2019). Although a clinician-based pain assessment has not been developed specifically for cancer pain in humans, behavioral observation scales produce similar results in nonverbal elderly adults with cancers and chronic pain (Passik et al., 2004; Bjoro & Herr, 2008); this supports the potential translational utility of the UFEPS/VET in cats with oral cancer. Both FORQ/CLIENT and the UFEPS/VET should undergo additional testing including repeating our work, readability testing and additional validity testing, including inter-rater reliability, responsiveness testing with other analgesics, further face validity testing and criterion validity testing.

We used an EVF and a COBO aesthesiometer to evaluate mechanical sensitivity. The EVF thresholds measured on the maxilla of healthy cats were variable; this could be due to the presence of highly sensitive tactile hairs of the vibrissal pad (Gogan et al., 1981; Grant et al., 2013). Even with that variability, we demonstrate here that cats with sublingual cancer were more sensitive to mechanical pressure near the mouth, and, interestingly at a distant anatomic site. Increased sensitivity around the mandibular region, maxilla, and eyes may be partly related to peripheral sensitization driven by locally produced nociceptor sensitizers, but part may also be driven by more central changes affecting multiple branches of the trigeminal nerve (Woolf, 2011; Vardeh & Naranjo, 2017). The three trigeminal branches (*i.e.,* ophthalmic, maxillary, and mandibular nerve) anatomically converge at the trigeminal ganglia, and signaling cross-talk is also possible at the brainstem (Walker, 1990). This pathway of signal transduction explains the maxillary and corneal hypersensitivity we observed. Peripheral sensitization may be due to inflammation, tumor cells infiltrating into the nerve fibers or other tumor-related factors (trauma, acidification, etc.) that increases the responsiveness and activity of nociceptors (Vardeh & Naranjo, 2017). The finding that cats with sublingual SCC had relatively low nociceptive thresholds at their metacarpus (in the absence of a tumor or other injury on their paw) may indicate central sensitization, or a generalized peripheral sensitization (Woolf, 2011; Vardeh & Naranjo, 2017). This is consistent with the finding that mice with experimentally-induced tongue SCC display widespread somatosensory sensitization (Ye et al., 2017).

The QST measures that were studied herein were able to distinguish healthy cats and cats with sublingual cancer. However, it must be remembered that QST results are likely affected by the “pain history”, underlying chronic pain, and stress. To address such potential bias, we performed a subset analysis in age-matched cats that came to the veterinary hospital for regular check-ups and/or vaccination, rather than simply visiting to participate in this study. This analysis supports our conclusion that the observed hypersensitivities were related to oral cancer. To that end, it is important to reflect upon the low ICC values reported herein for both mQST assays. Here, the lack of stability over time is not surprising given the small sample size, and this result is also similar to what has been reported for other forms of QST utilized in companion animals (Knazovicky et al., 2017). Thus, given our existing knowledge, it is important to consider that a well-designed experiment utilizing these assays must include carefully chosen concurrent controls.

To understand how well these QST data recapitulate the human oral cancer pain condition would require comparing our results with QST data from affected people. However, QST has been primarily used in human cancer patients to characterize the somatosensory changes that come with chemotherapy-induced peripheral neuropathy; data on OSCC related pain are lacking (Martland et al., 2020). Similar to the human condition is that pain is often restricted to the local tumor site. Dissimilar is that we measured spontaneous pain, whereas human oral SCC patient seem to report pain most frequently as having been evoked by such activities as eating and speaking.

As an initial evaluation of whether the methods used in the present study are able to detect reduced cancer pain after analgesic treatment, we performed assessments before and after administration of buprenorphine. Buprenorphine is an opioid that has been primarily used in cats for acute surgical pain (Steagall et al., 2009). Oral transmucosal administration has been shown to relieve pain and the maximum plasma concentration occurs about 30 min after application (Robertson et al., 2005; Stathopoulou et al., 2018).While buprenorphine may cause sedation, which could potentially confound our results, we saw significantly reduced sensitivities at both of the ipsilateral intermandibular test sites, and on the corneal surface after buprenorphine administration. This suggests that the instruments described herein are responsive to, and appear able to measure the effectiveness of analgesic interventions. Future validation studies should seek to understand whether the assays are also responsive to other analgesics. For example, it would be logical to test a non-steroidal anti-inflammatory drug; that was not done here due to concern for renal injury, which was heighted by the fact that after these pain measurements were made, each cat underwent general anesthesia for cancer treatment.

There are several limitations to the pilot work presented herein. First, while control cats did undergo thorough physical examination by a trained veterinarian, sedated oral examination and dental radiography were not performed, thus it is uncertain whether these cats were free of any dental disease. Second, while physical restraint for mQST was gentle and minimal, that handling and the environment (a hospital setting) could have affected nocifensive (behavioral) responses. Third, in Experiment 1, the amount of time between measurements (no less than 6-12 h) may have been too short; that interval was chosen because it was viewed as a balance between allowing time between tests while also minimizing the amount of hospitalization required for each cat. That latter point is an important consideration since clinic/hospital visits are potentially stressful to the animal, and that stress could influence test results. Nonetheless, is possible that during the second assessment, the observer may have been influenced by memory of answers to the first assessment. Strategies to reduce such potential bias must be considered for future validation work. Finally, in Experiment 2, our ability to rigorously assess discriminatory validity is compromised by failure to effectively mask the observer to experimental group; therefore, construct validity and responsiveness are subject to bias. Unfortunately, the frequency with which oral cancer afflicted cats display overt clinical signs of disease that are evident even to non-expert observers is high, and thus effective masking of well-trained researchers is viewed by these authors as being an unavoidable limitation of the work. Next steps in validation of these assays will be to larger scale studies, with incorporation of multiple observers such that we can rigorously assess inter-rater reliability.

## Conclusions

Together, the methods described in this manuscript can be used to provide what we believe is a comprehensive assessment of orofacial pain phenotypes in cats with oral cancer. If these instruments are to be successfully used for rigorous assessment of cancer-related pain and/or analgesic efficacy, additional large-scale inter-institutional studies should be performed to ensure that reproducible results are achievable. It would also be ideal to understand assay responses in the setting of various cancer treatments (*e.g.*, radiotherapy, chemotherapy, and surgery). Another important finding was that cats appear to have pain at the local/primary tumor site, and also have evidence of widespread somatosensory sensitization. Future work should aim to: (1) reaffirm this finding, (2) more comprehensively characterize the widespread changes and their mechanistic underpinnings, and (3) determine whether similar changes occur in humans, particularly in the absence of distant metastatic disease.

## Supplemental Information

10.7717/peerj.11984/supp-1 Supplemental Information 1 Test and re-test (test 1 and test 2) of MCPS-FOSCC, EVF, and COBO aesthesiometer of healthy and cancer cats

10.7717/peerj.11984/supp-2 Supplemental Information 2 Scores of Feline oral cancer Owner-Reported quality of life Questionnaire and Visual analogue score of healthy and cancer cats

10.7717/peerj.11984/supp-3 Supplemental Information 3 MCPS-FOSCC scores for healthy and cancer cats

10.7717/peerj.11984/supp-4 Supplemental Information 4 Electronic von Frey and Cochet-Bonnet aesthesiometer tests results of healthy and cancer cats

10.7717/peerj.11984/supp-5 Supplemental Information 5 Raw data for responsiveness validity testing

10.7717/peerj.11984/supp-6 Supplemental Information 6 Signalment of study cats

10.7717/peerj.11984/supp-7 Supplemental Information 7 Changes made to convert the canine owner-reported QOL questionnaire into an instrument that could be usable for cats with oral cancer There are 17 question items in the published canine owner-reported QOL questionnaire (CORQ) and each was scored on an 8-point scale (0-7). We rearranged the order of the questions to fit into four categories: behavior, activity, interaction, and orofacial discomfort. We changed the presentation of the items from how they are presented in CORQ; each item in preFORQ consists of a descriptive statement of an observable behavior and then the respondent is asked to indicate: (1) whether that behavior has been seen in the last week; (2) how many days within the past week their pet showed that particular behavior; and (3) how severe the signs were. In preFORQ, we removed the item “treatment interfered with his/her enjoyment of life” because not all cats in this study underwent cancer treatment. The evaluation of playfulness was moved into the question about activity, and so was “whether the cat does what he/she likes”. We added questions about mood, vocalization when at rest, ability to maintain normal hygiene, drinking and ability to position for normal urination and defecation. In the orofacial discomfort category, we included questions about “excessive drooling”, “difficulty eating normal food”, “trouble eating soft food”, “trouble resting their head down”, “discomfort or pain near mouth”, and “defensiveness when head was touched”. We believed these questions may be important in evaluating cats with oral cancers.

10.7717/peerj.11984/supp-8 Supplemental Information 8 Test –retest reliability analysis of EVF measurement and COBO aesthesiometer Anatomic location of the four measurement sites at which EVF measurements were made are indicated using black arrows on this representative image of a healthy cat: (A) right intermandibular space, (B) left intermandibular space, (C) right maxilla, (D) right metacarpus.

10.7717/peerj.11984/supp-9 Supplemental Information 9 Reliability test of EVF in healthy control cats Test –retest reliability analysis of EVF measurements and COBO aesthesiometer test results. The Bland-Altman plot (left) and the correlation (right) between test 1 and test 2 are presented (*P* = 0.0008, 0.0004, 0.0172, 0.0365 on right and left intermandibular space, right maxilla and right metacarpus, respectively). Each dot represents an individual cat. Open dots represent healthy control cats; closed dots denote cats with sublingual SCC.

10.7717/peerj.11984/supp-10 Supplemental Information 10 Reliability testing of EVF in cats with sublingual SCC Test –retest reliability assessment for Cochet-Bonnet aesthesiometer (cornea touch threshold, CTT) measurements. (A) The Bland-Altman plot and (B) the correlation between test 1 and test 2 are presented (*P* = 0.1823). Each dot represents an individual cat. Open dots denote healthy control cats; closed dots denote cats with sublingual SCC.

10.7717/peerj.11984/supp-11 Supplemental Information 11 Heatmap of frequency and severity results for the FORQ/CLIENT The averaged intensity of the observations with regard to (A) frequency and (B) severity. The question identification numbers are listed in Table S1 . The most frequent observations were excessive drooling, and trouble eating normal food; with regard to severity, the highest mean score (*vs.* other questions) was excessive drooling.

10.7717/peerj.11984/supp-12 Supplemental Information 12 Sex effects in QST assays Comparison of mechanical QST between male and female healthy cats. **(A-D)** EVF and (E) COBO aesthesiometer measurements showed that there was no significant difference of mean ±SEM between male (*n* = 6) and female (*n* = 10) healthy cats.

10.7717/peerj.11984/supp-13 Supplemental Information 13 Age-matched comparisons of various outcome measurements Cancer cats had significantly worse QOL and pain scores (*vs.* aged healthy cats) in terms of (A) preFORQ/CLIENT (B) VAS and (C) UFEPS/VET; Mann–Whitney test. Cancer cats also showed significantly decreased sensory thresholds in **(D-G)** EVF and **(H)** COBO aesthesiometer tests; unpaired *t-* test. Each dot represents the measurement of individual cats. Values from aged healthy cats are denoted by triangular shaped dots; closed/solid dots represent cats with sublingual SCC; ^∗∗^*P* < 0.01, ^∗∗∗^*P* < 0.001, * ^∗∗∗^*P* < 0.0001. All error bars depict SEM.

10.7717/peerj.11984/supp-14 Supplemental Information 14 Preliminary feline owner-reported orofacial cancer pain quality of life questionnaire (preFORQ)

10.7717/peerj.11984/supp-15 Supplemental Information 15 Modified Botucatu Multifactorial Composite Pain Scale –Adapted for Feline Oral Squamous Cell Carcinoma (UFEPS/VET)

10.7717/peerj.11984/supp-16 Supplemental Information 16 Summary of demographics of cats with sublingual SCC (FOSCC) and healthy controls

10.7717/peerj.11984/supp-17 Supplemental Information 17 Feline oral cancer owner-reported quality of life questionnaire (FORQ) category scores: median ±SD (range)

10.7717/peerj.11984/supp-18 Supplemental Information 18 Median (range) scores for individual questions on the FORQ for healthy control and cats with sublingual SCC. For details of individual questions, see Table S1

10.7717/peerj.11984/supp-19 Supplemental Information 19 Baseline measures (Mean ±SD and range) in aged healthy control and cats with sublingual SCC (age, body weight, mechanical sensory threshold [MST]) and cornea touch threshold [CTT])

10.7717/peerj.11984/supp-20 Supplemental Information 20 Outcome measure values (Mean ±SD and range) prior to buprenorphine administration (Test 1), after buprenorphine administration (BUP), and the following day (Test 2) The statistical analysis of MCPS-FOSCC was performed by Wilcoxon matched-pairs signed rank test; MST and CCT were analyzed by paired *t*-test.

## Additional Information and Declarations

### Data Availability

The following information was supplied regarding data availability: Data for the FORQ, the revised FORQ, and data from healthy cats are available in the Supplemental Files.
