# Supplementary material for: Behavioral phenotyping of cancer pain in domesticated cats with naturally occurring squamous cell carcinoma of the tongue: initial validation studies provide evidence for regional and widespread algoplasticity
Source: PeerJ. 2021 Aug 16;9:e11984. doi: 10.7717/peerj.11984 (PMC8375511; doi:10.7717/peerj.11984)
Supplement: Supplemental Information 7 — There are 17 question items in the published canine owner-reported QOL questionnaire (CORQ) and each was scored on an 8-point scale (0-7). We rearranged the order of the questions to fit into four categories: behavior, activity, interaction, and orofacial discomfort. We changed the presentation of the items from how they are presented in CORQ; each item in preFORQ consists of a descriptive statement of an observable behavior and then the respondent is asked to indicate: (1) whether that behavior has been seen in the last week; (2) how many days within the past week their pet showed that particular behavior; and (3) how severe the signs were. In preFORQ, we removed the item “treatment interfered with his/her enjoyment of life” because not all cats in this study underwent cancer treatment. The evaluation of playfulness was moved into the question about activity, and so was “whether the cat does what he/she likes”. We added questions about mood, vocalization when at rest, ability to maintain normal hygiene, drinking and ability to position for normal urination and defecation. In the orofacial discomfort category, we included questions about “excessive drooling”, “difficulty eating normal food”, “trouble eating soft food”, “trouble resting their head down”, “discomfort or pain near mouth”, and “defensiveness when head was touched”. We believed these questions may be important in evaluating cats with oral cancers. [file peerj-09-11984-s007.pdf]

Supplemental Article 1.

Preliminary FORQ adaptation

There are 17 question items in the published canine owner-reported QOL questionnaire (CORQ) and each was scored on an 8-point scale (0-7). We rearranged the order of the questions to fit into four categories: behavior, activity, interaction, and orofacial discomfort. We changed the presentation of the items from how they are presented in CORQ; each item in preFORQ consists of a descriptive statement of an observable behavior and then the respondent is asked to indicate: (1) whether that behavior has been seen in the last week; (2) how many days within the past week their pet showed that particular behavior; and (3) how severe the signs were. In preFORQ, we removed the item "treatment interfered with his/her enjoyment of life" because not all cats in this study underwent cancer treatment. The evaluation of playfulness was moved into the question about activity, and so was "whether the cat does what he/she likes". We added questions about mood, vocalization when at rest, ability to maintain normal hygiene, drinking and ability to position for normal urination and defecation. In the orofacial discomfort category, we included questions about "excessive drooling", "difficulty eating normal food", "trouble eating soft food", "trouble resting their head down", "discomfort or pain near mouth", and "defensiveness when head was touched". We believed these questions may be important in evaluating cats with oral cancers.
