# Supplementary figures and images for: Behavioral phenotyping of cancer pain in domesticated cats with naturally occurring squamous cell carcinoma of the tongue: initial validation studies provide evidence for regional and widespread algoplasticity
Source: PeerJ. 2021 Aug 16;9:e11984. doi: 10.7717/peerj.11984 (PMC8375511; doi:10.7717/peerj.11984)

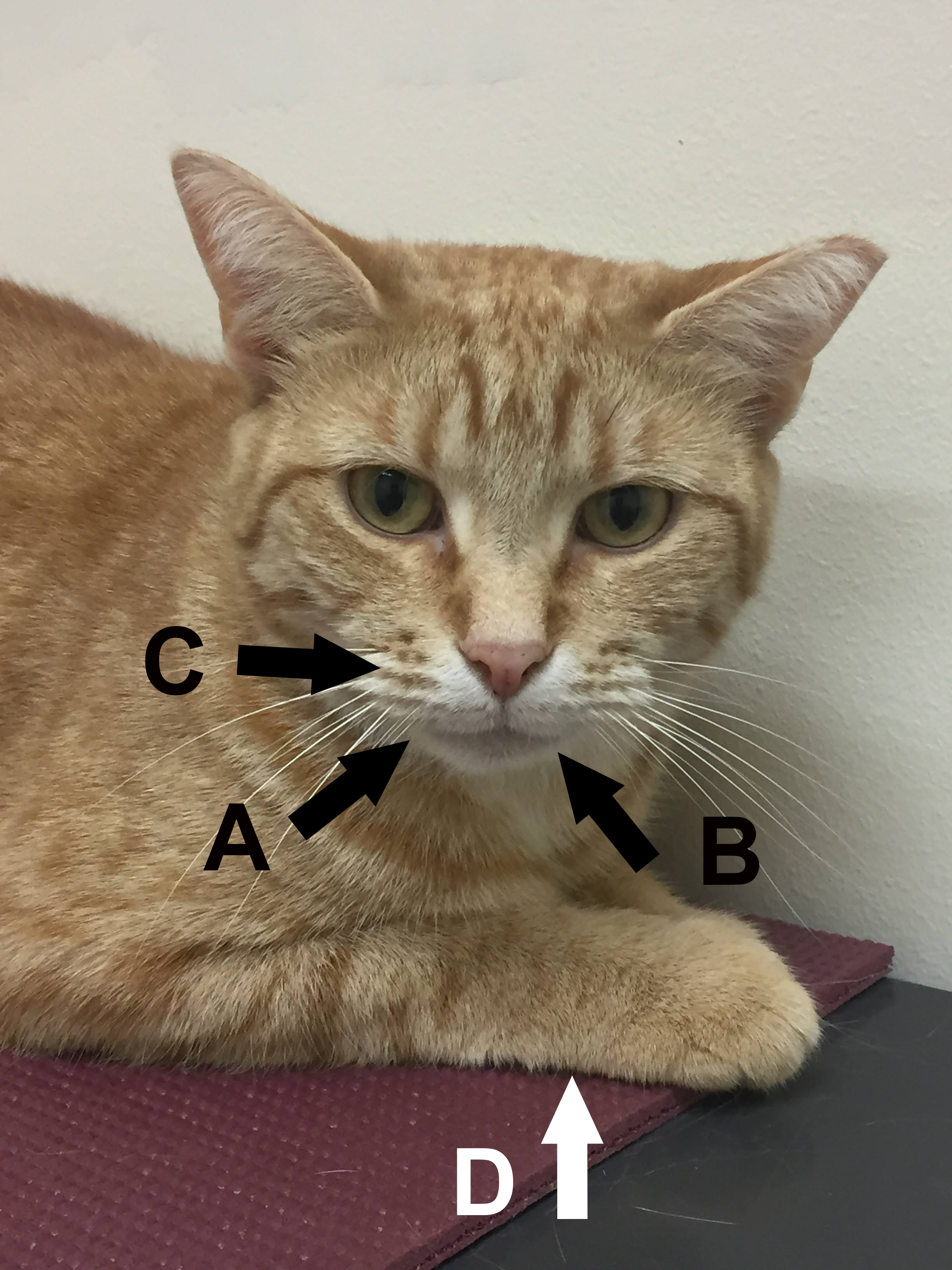

Supplement: Supplemental Information 8 — Anatomic location of the four measurement sites at which EVF measurements were made are indicated using black arrows on this representative image of a healthy cat: (A) right intermandibular space, (B) left intermandibular space, (C) right maxilla, (D) right metacarpus. [file peerj-09-11984-s008.png]

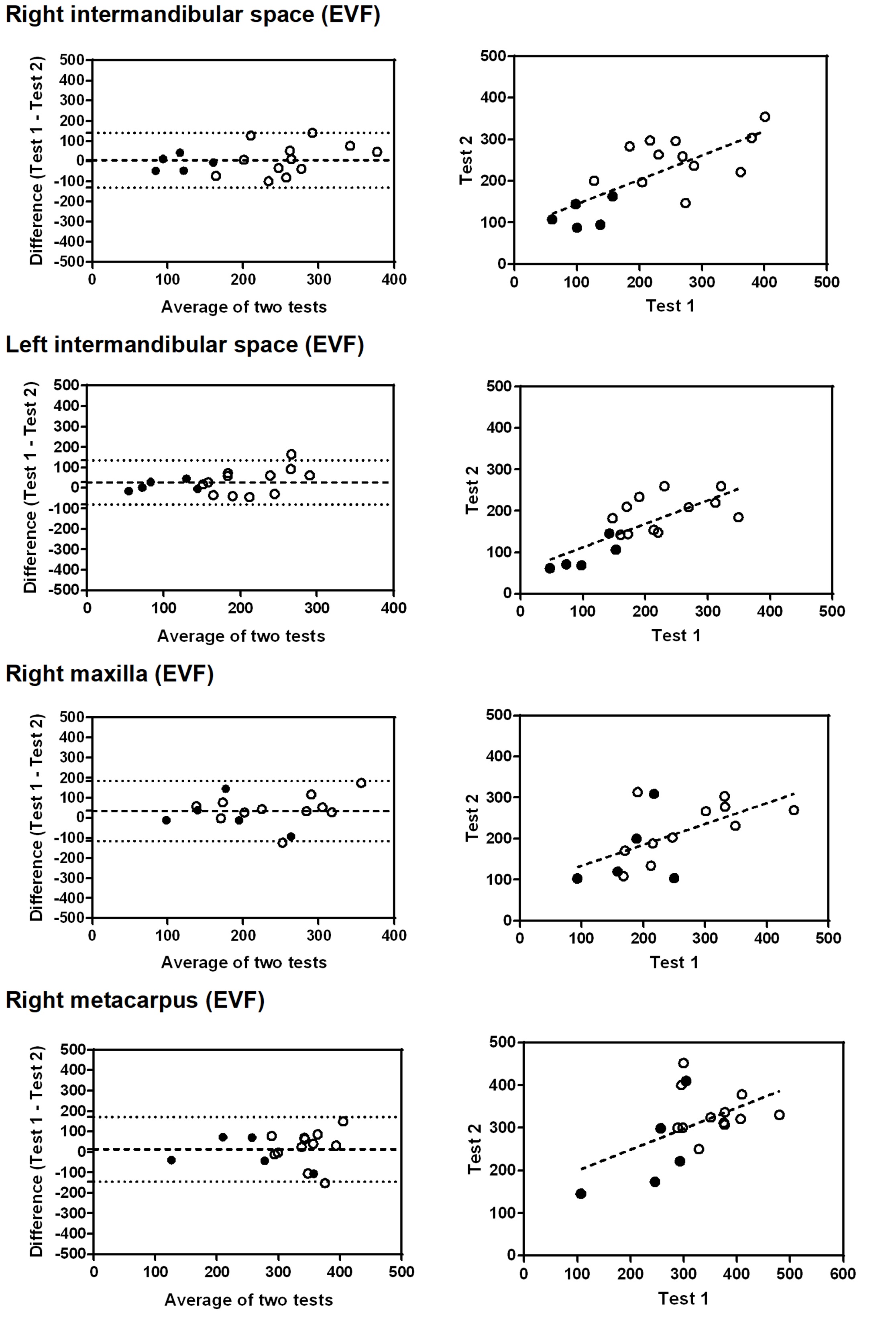

Supplement: Supplemental Information 9 — Test –retest reliability analysis of EVF measurements and COBO aesthesiometer test results. The Bland-Altman plot (left) and the correlation (right) between test 1 and test 2 are presented (P = 0.0008, 0.0004, 0.0172, 0.0365 on right and left intermandibular space, right maxilla and right metacarpus, respectively). Each dot represents an individual cat. Open dots represent healthy control cats; closed dots denote cats with sublingual SCC. [file peerj-09-11984-s009.jpg]

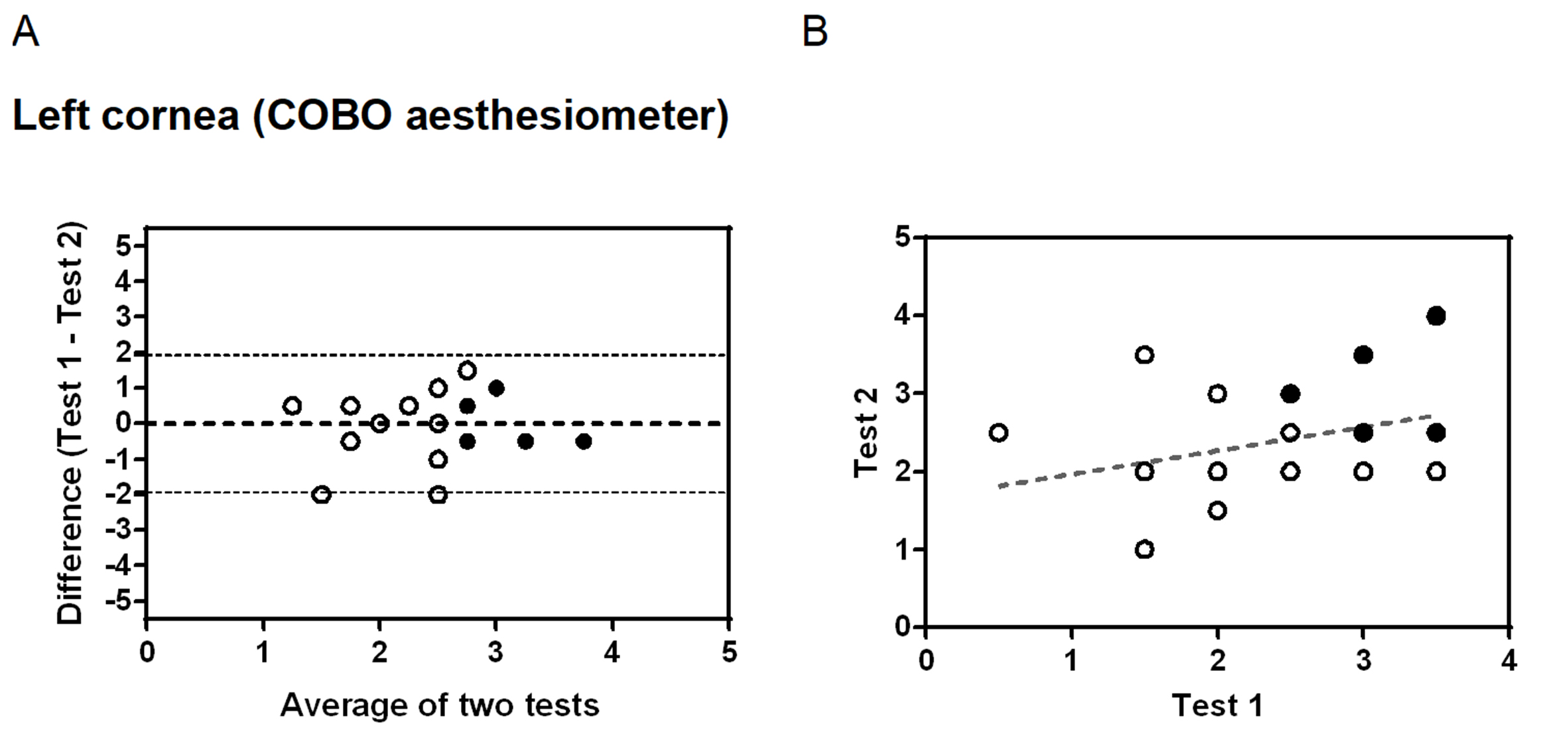

Supplement: Supplemental Information 10 — Test –retest reliability assessment for Cochet-Bonnet aesthesiometer (cornea touch threshold, CTT) measurements. (A) The Bland-Altman plot and (B) the correlation between test 1 and test 2 are presented (P = 0.1823). Each dot represents an individual cat. Open dots denote healthy control cats; closed dots denote cats with sublingual SCC. [file peerj-09-11984-s010.jpg]

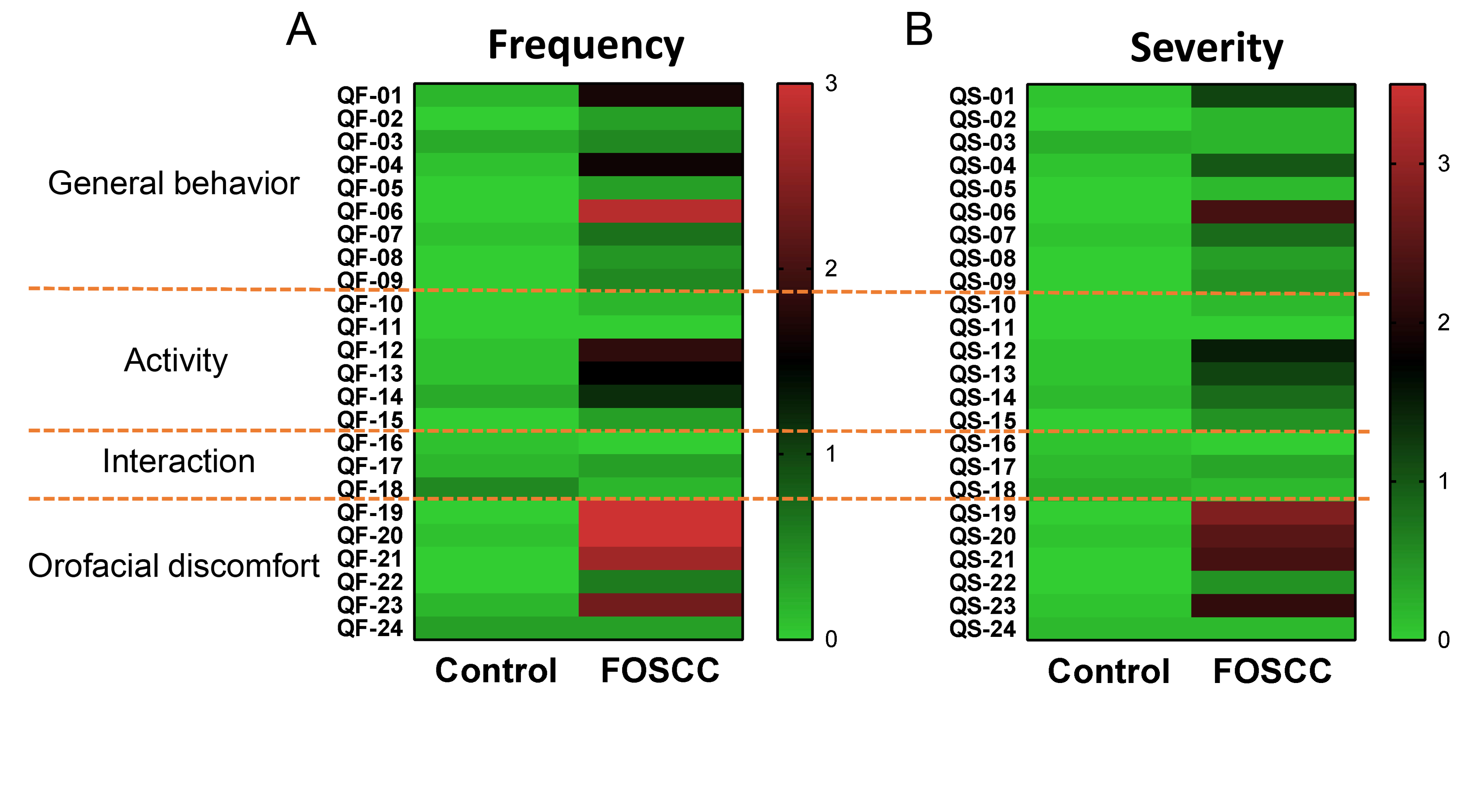

Supplement: Supplemental Information 11 — The averaged intensity of the observations with regard to (A) frequency and (B) severity. The question identification numbers are listed in Table S1 . The most frequent observations were excessive drooling, and trouble eating normal food; with regard to severity, the highest mean score (vs. other questions) was excessive drooling. [file peerj-09-11984-s011.png]

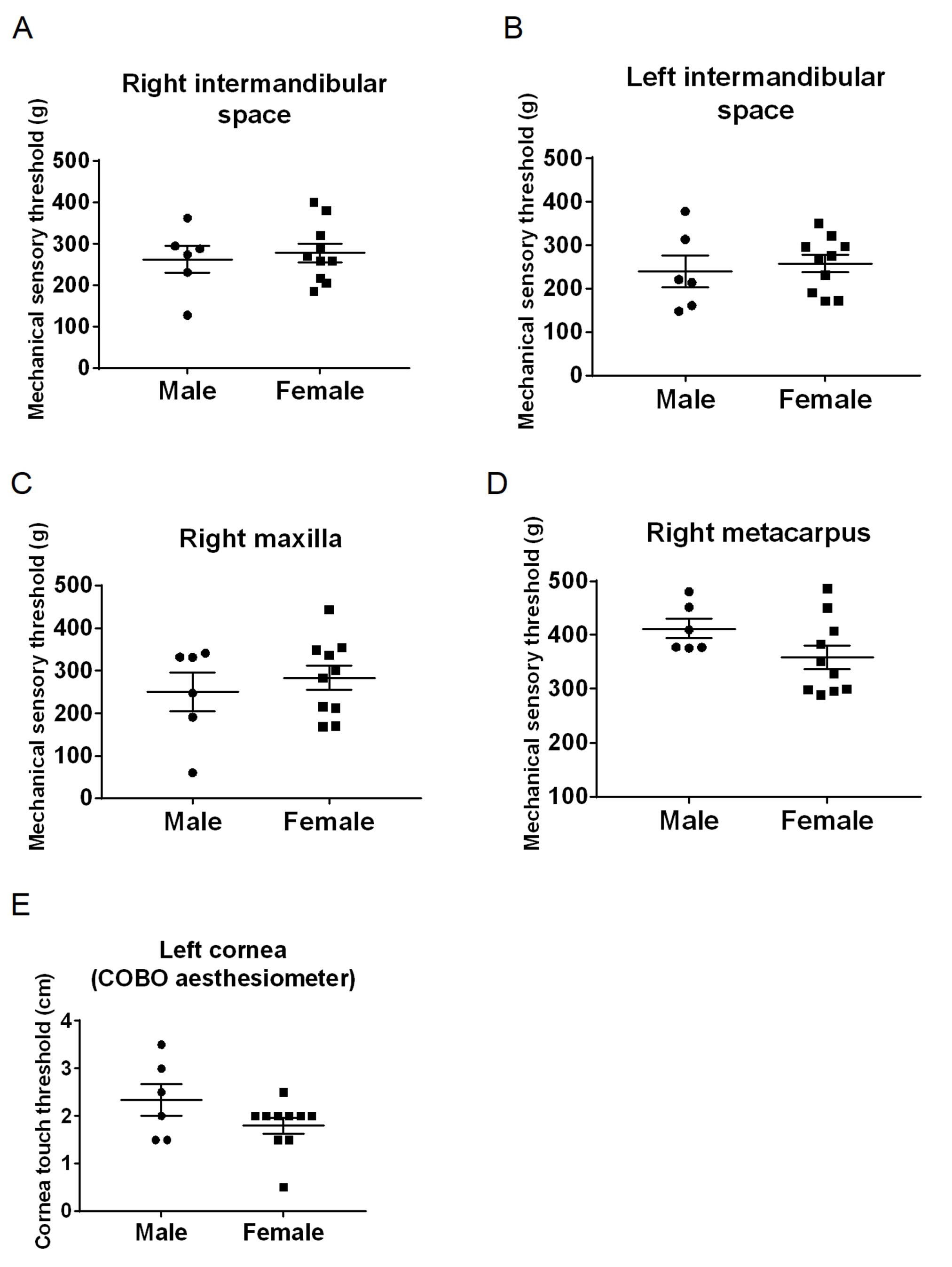

Supplement: Supplemental Information 12 — Comparison of mechanical QST between male and female healthy cats. (A-D) EVF and (E) COBO aesthesiometer measurements showed that there was no significant difference of mean ±SEM between male (n = 6) and female (n = 10) healthy cats. [file peerj-09-11984-s012.jpg]

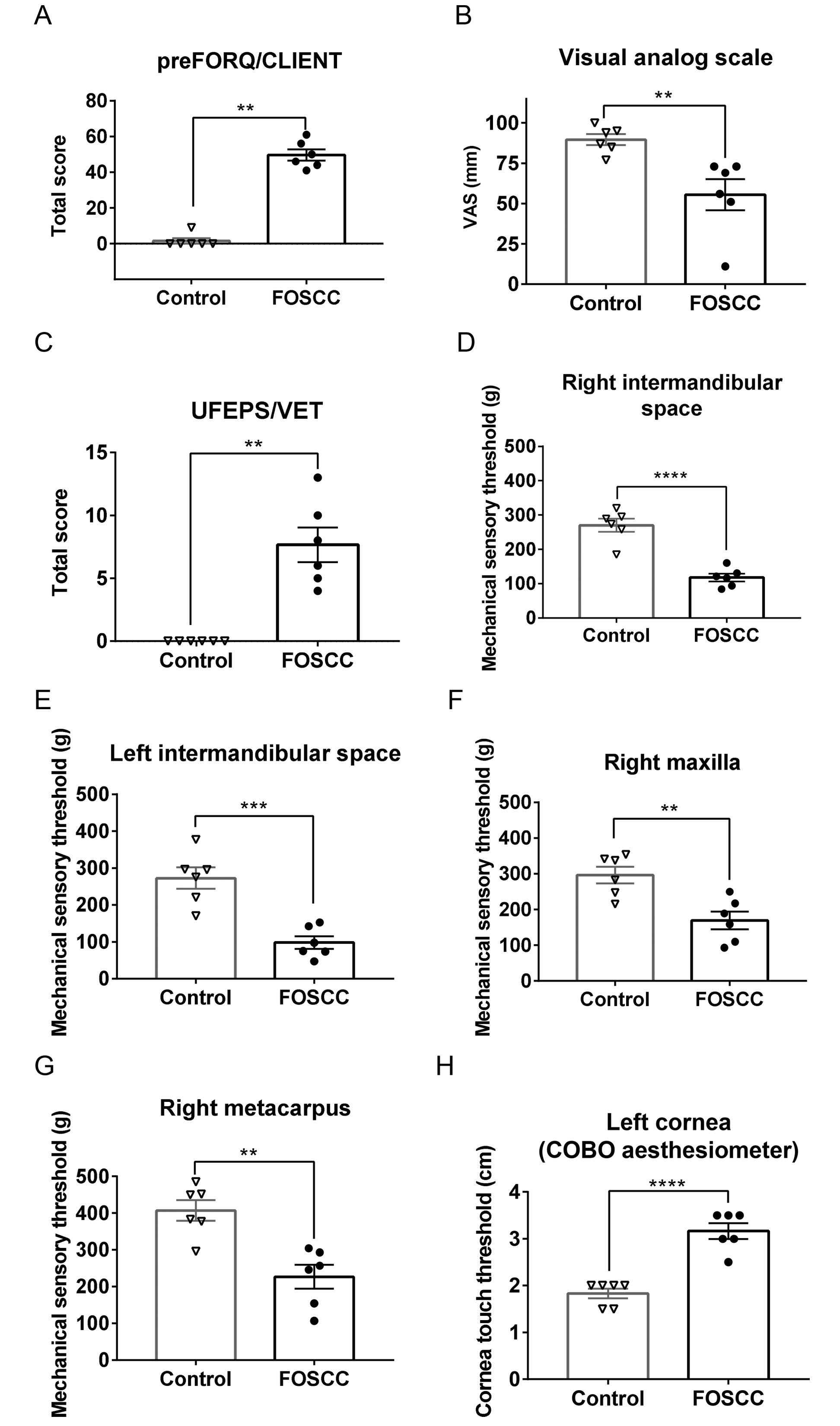

Supplement: Supplemental Information 13 — Cancer cats had significantly worse QOL and pain scores (vs. aged healthy cats) in terms of (A) preFORQ/CLIENT (B) VAS and (C) UFEPS/VET; Mann–Whitney test. Cancer cats also showed significantly decreased sensory thresholds in (D-G) EVF and (H) COBO aesthesiometer tests; unpaired t- test. Each dot represents the measurement of individual cats. Values from aged healthy cats are denoted by triangular shaped dots; closed/solid dots represent cats with sublingual SCC; ∗∗P < 0.01, ∗∗∗P < 0.001, * ∗∗∗P < 0.0001. All error bars depict SEM. [file peerj-09-11984-s013.jpg]
