# Supplementary material for: Behavioral phenotyping of cancer pain in domesticated cats with naturally occurring squamous cell carcinoma of the tongue: initial validation studies provide evidence for regional and widespread algoplasticity
Source: PeerJ. 2021 Aug 16;9:e11984. doi: 10.7717/peerj.11984 (PMC8375511; doi:10.7717/peerj.11984)
Supplement: Supplemental Information 14 [file peerj-09-11984-s014.docx]

**Supplemental Table S1.** Preliminary feline owner-reported orofacial cancer pain quality of life questionnaire (preFORQ/CLIENT)

| During the past 7 days, my cat: | No | If YES,  ***how often*** did your cat have it? | | | | If YES,  ***how severe*** was it usually? | | | |
| --- | --- | --- | --- | --- | --- | --- | --- | --- | --- |
|  |  | Rarely | Sometimes | Usually | Always | Mild | Moderate | Severe | Very severe |
| **Behavior** |  |  |  |  |  |  |  |  |  |
| 1. Had low energy? | ⭘ | ⭘ | ⭘ | ⭘ | ⭘ | ⭘ | ⭘ | ⭘ | ⭘ |
| 1. Was reluctant to wake up? | ⭘ | ⭘ | ⭘ | ⭘ | ⭘ | ⭘ | ⭘ | ⭘ | ⭘ |
| 1. Had altered mood? | ⭘ | ⭘ | ⭘ | ⭘ | ⭘ | ⭘ | ⭘ | ⭘ | ⭘ |
| 1. Had trouble getting comfortable? | ⭘ | ⭘ | ⭘ | ⭘ | ⭘ | ⭘ | ⭘ | ⭘ | ⭘ |
| 1. Growled or groaned when resting? | ⭘ | ⭘ | ⭘ | ⭘ | ⭘ | ⭘ | ⭘ | ⭘ | ⭘ |
| 1. Could not maintain hygiene (i.e., grooming)? | ⭘ | ⭘ | ⭘ | ⭘ | ⭘ | ⭘ | ⭘ | ⭘ | ⭘ |
| 1. Had decreased appetite? | ⭘ | ⭘ | ⭘ | ⭘ | ⭘ | ⭘ | ⭘ | ⭘ | ⭘ |
| 1. Drank less water than usual? | ⭘ | ⭘ | ⭘ | ⭘ | ⭘ | ⭘ | ⭘ | ⭘ | ⭘ |
| 1. Had trouble positioning to defecate/urinate? | ⭘ | ⭘ | ⭘ | ⭘ | ⭘ | ⭘ | ⭘ | ⭘ | ⭘ |
| **Activity** |  |  |  |  |  |  |  |  |  |
| 1. Had trouble with mobility? | ⭘ | ⭘ | ⭘ | ⭘ | ⭘ | ⭘ | ⭘ | ⭘ | ⭘ |
| 1. Fell or lost balance? | ⭘ | ⭘ | ⭘ | ⭘ | ⭘ | ⭘ | ⭘ | ⭘ | ⭘ |
| 1. Did not do what he/she likes (e.g. chasing, playing, etc.)? | ⭘ | ⭘ | ⭘ | ⭘ | ⭘ | ⭘ | ⭘ | ⭘ | ⭘ |
| 1. Did not act like his/her normal self? | ⭘ | ⭘ | ⭘ | ⭘ | ⭘ | ⭘ | ⭘ | ⭘ | ⭘ |
| 1. Had decreased enjoyment of life? | ⭘ | ⭘ | ⭘ | ⭘ | ⭘ | ⭘ | ⭘ | ⭘ | ⭘ |
| 1. Did not sleep well? | ⭘ | ⭘ | ⭘ | ⭘ | ⭘ | ⭘ | ⭘ | ⭘ | ⭘ |
| **Interaction** |  |  |  |  |  |  |  |  |  |
| 1. Was unwilling to be near me? | ⭘ | ⭘ | ⭘ | ⭘ | ⭘ | ⭘ | ⭘ | ⭘ | ⭘ |
| 1. Showed a decreased amount of affection? | ⭘ | ⭘ | ⭘ | ⭘ | ⭘ | ⭘ | ⭘ | ⭘ | ⭘ |
| 1. Did not like to be pet or touched? | ⭘ | ⭘ | ⭘ | ⭘ | ⭘ | ⭘ | ⭘ | ⭘ | ⭘ |
| **Oral/facial discomfort** |  |  |  |  |  |  |  |  |  |
| 1. Had excessive drooling? | ⭘ | ⭘ | ⭘ | ⭘ | ⭘ | ⭘ | ⭘ | ⭘ | ⭘ |
| 1. Had difficulty eating his/her normal food? | ⭘ | ⭘ | ⭘ | ⭘ | ⭘ | ⭘ | ⭘ | ⭘ | ⭘ |
| 1. Was offered and had trouble eating soft food? | ⭘ | ⭘ | ⭘ | ⭘ | ⭘ | ⭘ | ⭘ | ⭘ | ⭘ |
| 1. Had trouble lying down his/her head? | ⭘ | ⭘ | ⭘ | ⭘ | ⭘ | ⭘ | ⭘ | ⭘ | ⭘ |
| 1. Felt discomfort or pain near the mouth? | ⭘ | ⭘ | ⭘ | ⭘ | ⭘ | ⭘ | ⭘ | ⭘ | ⭘ |
| 1. Was defensive when their head was touched? | ⭘ | ⭘ | ⭘ | ⭘ | ⭘ | ⭘ | ⭘ | ⭘ | ⭘ |

For this final question, please mark an ***X*** along the line to show your cat’s overall ***current*** quality of life

|-----------------------------------------------------------------------------------|

Worst Imaginable Quality of Life Perfect Quality of Life
