# Supplementary material for: Behavioral phenotyping of cancer pain in domesticated cats with naturally occurring squamous cell carcinoma of the tongue: initial validation studies provide evidence for regional and widespread algoplasticity
Source: PeerJ. 2021 Aug 16;9:e11984. doi: 10.7717/peerj.11984 (PMC8375511; doi:10.7717/peerj.11984)
Supplement: Supplemental Information 15 [file peerj-09-11984-s015.docx]

**Supplemental Table S2.** Modified Botucatu Multidimensional Composite Pain Scale – Adapted for Feline Oral Squamous Cell Carcinoma (UFEPS/VET). The changes were indicated in **bold**.

| Subscale 1: PAIN EXPRESSION (0-12) | | |
| --- | --- | --- |
| Miscellaneous  behavior | Observe and mark the presence of the behaviors listed below   1. - The cat is laying down and quiet, but moving its tail 2. - The cat contracts and extends its thoracic limbs and/or contracts its neck muscles 3. - The cat’s eyes are partially closed (eyes half closed) 4. - **The cat licks, has ptyalism, and/or chattering (jaw shakes)** |  |
|  | - All above behaviors are absent | 0 |
|  | - Presence of one of the above behaviors | 1 |
|  | - Presence of two of the above behaviors | 2 |
|  | - Presence of three of the above behaviors | 3 |
| Reaction to palpation of the area around the mouth cavity | - The cat does not react when the **mouth** is touched or pressed | 0 |
|  | - The cat does not react when the area around the **mouth** is touched, but does react when it is touched and pressed. It may vocalize and/or try to bite | 1 |
|  | - The cat reacts when the **mouth** is touched and when pressed. It may vocalize and/or try to bite. | 2 |
|  | - The cat reacts when the observer approaches the **mouth**. It may vocalize and/or try to bite. The cat does not allow palpation around mouth cavity. | 3 |
| Reaction to palpation of the **head** | - The cat does not react when the **head** is touched | 0 |
|  | - The cat does not react when the **head and neck** are touched, but does react when it is pressed. The **neck** is tense | 1 |
|  | - The cat reacts when the **head and neck** are touched and when pressed. The **neck** is tense | 2 |
|  | - The cat reacts when the observer approaches the **head**. It may vocalize and/or try to bite. The cat does not allow palpation of the **head and neck** | 3 |
| Vocalization | - The cat is quiet, purring when stimulated, or meows interacting with the observer, but does not growl, groan, or hiss | 0 |
|  | - The cat purrs spontaneously (without being stimulated or handled by the observer) | 1 |
|  | - The cat growls, howls, or hisses when handled by the observer (when its body position is changed by the observer) | 2 |
|  | - The cat growls, howls, hisses spontaneously (without being stimulated or handled by the observer) | 3 |
|  | Subtotal |  |
| Subscale 2: PSYCHOMOTOR CHANGE (0-12) | | |
| Posture | - The cat is in a natural posture with relaxed muscles (it moves normally) | 0 |
|  | - The cat is in a natural posture but is tense (it moves little or is reluctant to move) | 1 |
|  | - The cat is sitting or in sternal recumbency with its back arched and head down; or   The cat is in dorso-lateral recumbency with its pelvic limbs extended or contracted | 2 |
|  | - The cat frequently alters its body position in an attempt to find a comfortable posture | 3 |
| Comfort | - The cat is comfortable, awake or asleep, and interacts when stimulated (it interacts with the observer and/or is interested in its surroundings) | 0 |
|  | - The cat is quiet and slightly receptive when stimulated (it interacts little with the observer and/or is not very interested in its surroundings) | 1 |
|  | - The cat is quiet and “dissociated from the environment” (even when stimulated it does not interact with the observer and/or has no interest in its surroundings)   The cat may be facing the back of the cage | 2 |
|  | - The cat is uncomfortable, restless (frequently changes its body position), and slightly receptive when stimulated or “dissociated from the environment”   The cat may be facing the back of the cage | 3 |
| Activity | - The cat moves normally (it immediately moves when the cage is opened; outside the cage it moves spontaneously when stimulated or handled) | 0 |
|  | - The cat moves more than normal (inside the cage it moves continuously from side to side) | 1 |
|  | - The cat is quieter than normal (it may hesitate to leave the cage and if removed from the cage tends to return, outside the cage it moves a little after stimulation or handling) | 2 |
|  | - The cat is reluctant to move (it may hesitate to leave the cage and if removed from the cage tends to return, outside the cage it does not move even when stimulated or handled) | 3 |
| Attitude | Observe and mark the presence of the mental states listed below   1. Satisfied: The cat is alert and interested in its surroundings (explores its surroundings), friendly and interactive with the observer (plays and/or responds to stimuli)  - *The cat may initially interact with the observer through games to distract it from the pain. Carefully observe to distinguish between distraction and satisfaction games*  1. Uninterested: The cat does not interact with the observer (not interested by toys or plays a little; does not respond to calls or strokes from the observer)  - *In cats, which don’t like to play, evaluate interaction with the observer by its response to calls and strokes*  1. Indifferent: The cat is not interested in its surroundings (it is not curious; it does not explore its surroundings)  - *The cat can initially be afraid to explore its surroundings. The observer needs to handle the cat and encourage it to move itself (take it out of the cage and/or change its body position)*  1. Anxious: The cat is frightened (it tries to hide or escape) or nervous (demonstrating impatience and growling, howling, or hissing when stroked and/or handled) 2. Aggressive: The cat is aggressive (tries to bite or scratch when stroked or handled) |  |
|  | - Presence of the mental state A | 0 |
|  | - Presence of one of the mental states B, C, D, or E | 1 |
|  | - Presence of two of the mental states B, C, D, or E | 2 |
|  | - Presence of three or all of the mental states B, C, D, or E | 3 |
|  | Subtotal |  |
| **Subscale 3: PHYSIOLOGICAL VARIABLES (0-3)** | | |
| Appetite | - The cat is eating normally | 0 |
|  | - The cat is eating more than normal | 1 |
|  | - The cat is eating less than normal | 2 |
|  | - The cat is not interested in food | 3 |
|  | Subtotal |  |
|  | **Total Score** |  |
