# Supplementary material for: Behavioral phenotyping of cancer pain in domesticated cats with naturally occurring squamous cell carcinoma of the tongue: initial validation studies provide evidence for regional and widespread algoplasticity
Source: PeerJ. 2021 Aug 16;9:e11984. doi: 10.7717/peerj.11984 (PMC8375511; doi:10.7717/peerj.11984)
Supplement: Supplemental Information 16 [file peerj-09-11984-s016.docx]

**Supplemental Table S3.** Summary of demographics of cats with sublingual SCC (FOSCC) and healthy controls.

|  | Control (n = 16) | FOSCC (n = 6) | All (n = 22) |
| --- | --- | --- | --- |
| Sex  Male - castrated  Female - spayed | 6  10 | 2  4 | 8  14 |
| Age (years)  Median  Range (min-max)  Mean  SD  SEM | 5  1.5-15  6.3  4.12  1.03 | 10  9-14  10.5  1.76  0.72 | 8  1.5-15  7.5  4.06  0.86 |
| Body weight (kg)  Median  Range (min-max)  Mean  SD  SEM | 4.47  3.3-5.3  4.45  0.63  0.16 | 3.41  3.05-4.6  3.66  0.58  0.23 | 4.17  3.05-5.3  4.23  0.70  0.15 |
| Breed  Domestic short hair  Domestic long hair  Bengal | 13  1  2 | 5  1  0 | 18  2  2 |
