# Supplementary material for: Behavioral phenotyping of cancer pain in domesticated cats with naturally occurring squamous cell carcinoma of the tongue: initial validation studies provide evidence for regional and widespread algoplasticity
Source: PeerJ. 2021 Aug 16;9:e11984. doi: 10.7717/peerj.11984 (PMC8375511; doi:10.7717/peerj.11984)
Supplement: Supplemental Information 17 [file peerj-09-11984-s017.docx]

Supplemental **Table S4.** Preliminary feline oral cancer owner-reported quality of life questionnaire (preFORQ/CLIENT) category scores: median ± SD (range).

| Question item | Frequency | | | Severity | | |
| --- | --- | --- | --- | --- | --- | --- |
|  | Control | FOSCC | *P*-value | Control | FOSCC | p-value |
| General behavior | 0 ± 1.03 (0-3) | 7 ± 3.33 (6-14) | **<0.0001** | 0 ± 0.89 (0-3) | 6.5 ± 1.86 (3-8) | **<0.0001** |
| Activity | 0 ± 0.87 (0-3) | 5 ± 2.37 (2-8) | **<0.0001** | 0 ± 0.77 (0-3) | 4 ± 2.64 (1-8) | **<0.0001** |
| Interaction | 0 ± 1.03 (0-3) | 0 ± 1.23 (0-3) | >0.9999 | 0 ± 0.72 (0-2) | 0 ± 1.23 (0-3) | >0.9999 |
| Orofacial discomfort | 0 ± 1.32 (0-5) | 13 ± 4.36 (6-16) | **<0.0001** | 0 ± 0.68 (0-2) | 12 ± 3.2 (6-13) | **<0.0001** |
| Subtotal | 0 ± 3.66 (0-11) | 24.5 ± 6.37 (18-37) | **<0.0001** | 0 ± 2.70 (0-9) | 21 ± 4.04 (17-28) | **<0.0001** |
