# Supplementary material for: Behavioral phenotyping of cancer pain in domesticated cats with naturally occurring squamous cell carcinoma of the tongue: initial validation studies provide evidence for regional and widespread algoplasticity
Source: PeerJ. 2021 Aug 16;9:e11984. doi: 10.7717/peerj.11984 (PMC8375511; doi:10.7717/peerj.11984)
Supplement: Supplemental Information 18 [file peerj-09-11984-s018.docx]

Supplemental **Table S5**. Median (range) scores for individual questions on the preFORQ/CLIENT for healthy control and cats with sublingual SCC. For details of individual questions, see **Table S1**.

| Question item | Frequency | | | Severity | | |
| --- | --- | --- | --- | --- | --- | --- |
|  | Control | FOSCC | *P*-value | Control | FOSCC | *P*-value |
| *General behavior* |  |  |  |  |  |  |
| Q-01 | 0 (0-2) | 2 (0-3) | **0.0011** | 0 (0-1) | 1 (0-2) | **0.0007** |
| Q-02 | 0 (0-0) | 0 (0-1) | 0.0649 | 0 (0-0) | 0 (0-1) | 0.2273 |
| Q-03 | 0 (0-2) | 0 (0-2) | 0.4997 | 0 (0-2) | 0 (0-1) | >0.9999 |
| Q-04 | 0 (0-1) | 2 (0-3) | **0.0016** | 0 (0-1) | 1 (0-2) | **0.0087** |
| Q-05 | 0 (0-0) | 0 (0-2) | 0.2727 | 0 (0-0) | 0 (0-1) | 0.2609 |
| Q-06 | 0 (0-0) | 3 (2-4) | **<0.0001** | 0 (0-0) | 2.5 (1-3) | **<0.0001** |
| Q-07 | 0 (0-1) | 0.5 (0-2) | 0.0349 | 0 (0-1) | 0.5 (0-2) | **0.0209** |
| Q-08 | 0 (0-0) | 0 (0-2) | 0.2381 | 0 (0-0) | 0 (0-2) | 0.2273 |
| Q-09 | 0 (0-0) | 0 (0-3) | 0.2727 | 0 (0-0) | 0 (0-3) | 0.2609 |
| *Activity* |  |  |  |  |  |  |
| Q-10 | 0 (0-0) | 0 (0-1) | 0.2727 | 0 (0-0) | 0 (0-1) | 0.2609 |
| Q-11 | 0 (0-0) | 0 (0-0) | >0.9999 | 0 (0-0) | 0 (0-0) | >0.9999 |
| Q-12 | 0 (0-1) | 2 (0-3) | **0.0004** | 0 (0-1) | 1 (0-3) | **0.0007** |
| Q-13 | 0 (0-1) | 2 (0-3) | **0.0021** | 0 (0-1) | 1.5 (0-2) | **0.0032** |
| Q-14 | 0 (0-2) | 0.5 (0-4) | 0.0549 | 0 (0-1) | 0.5 (0-2) | **0.032** |
| Q-15 | 0 (0-0) | 0 (0-1) | 0.0649 | 0 (0-0) | 0 (0-2) | 0.0593 |
| *Interaction* |  |  |  |  |  |  |
| Q-16 | 0 (0-1) | 0 (0-0) | >0.9999 | 0 (0-1) | 0 (0-0) | >0.9999 |
| Q-17 | 0 (0-1) | 0 (0-2) | 0.6883 | 0 (0-1) | 0 (0-2) | 0.6928 |
| Q-18 | 0 (0-3) | 0 (0-1) | 0.7703 | 0 (0-1) | 0 (0-1) | >0.9999 |
| *Orofacial discomfort* |  |  |  |  |  |  |
| Q-19 | 0 (0-0) | 3.5 (1-4) | **<0.0001** | 0 (0-0) | 3 (2-4) | **<0.0001** |
| Q-20 | 0 (0-1) | 3.5 (1-4) | **<0.0001** | 0 (0-1) | 2 (1-4) | **<0.0001** |
| Q-21 | 0 (0-0) | 3 (1-4) | **<0.0001** | 0 (0-0) | 2.5 (1-3) | **<0.0001** |
| Q-22 | 0 (0-0) | 0 (0-2) | 0.0649 | 0 (0-0) | 0 (0-2) | 0.0593 |
| Q-23 | 0 (0-2) | 2.5 (0-4) | **0.0007** | 0 (0-1) | 2 (0-4) | **0.0002** |
| Q-24 | 0 (0-3) | 0 (0-2) | 0.8442 | 0 (0-1) | 0 (0-1) | >0.9999 |
