# Supplementary material for: Behavioral phenotyping of cancer pain in domesticated cats with naturally occurring squamous cell carcinoma of the tongue: initial validation studies provide evidence for regional and widespread algoplasticity
Source: PeerJ. 2021 Aug 16;9:e11984. doi: 10.7717/peerj.11984 (PMC8375511; doi:10.7717/peerj.11984)
Supplement: Supplemental Information 19 [file peerj-09-11984-s019.docx]

Supplemental **Table S6.** Baseline measures (Mean ± SD and range) in aged healthy control and cats with sublingual SCC (age, body weight, mechanical sensory threshold [MST]) and cornea touch threshold [CTT]).

| Category | Aged control | FOSCC | Mean difference (95% CI) | *P*-value |
| --- | --- | --- | --- | --- |
| Age (years)  Body weight (kg)  MST (g) | 10.7 ± 3.1  4.7 ± 0.6 | 10.5 ± 1.8  3.7 ± 0.6 | -0.2 ± 1.4  (-3.4, 3.1)  -1.0 ± 0.3  (-1.8, -0.3) | 0.9106  **0.0106** |
| Right intermandibular space | 270.1 ± 46.7 | 117.7 ± 27.2 | -152.4 ± 22.1  (-201.6, -103.2) | **<0.0001** |
| Left intermandibular space | 272.9 ± 71.2 | 98.1 ± 41.7 | -174.8 ± 33.7  (-250, -100) | **0.0004** |
| Right maxilla | 296.3 ± 56.9 | 169.6 ± 61.1 | -126.7 ± 34.1  (-202.6, -50.8) | **0.004** |
| Right metacarpus | 407.2 ± 68.9 | 227 ± 79.2 | -180.2 ± 42.9  (-275.7, -84.7) | **0.0018** |
| CCT (cm) | 1.8 ± 0.3 | 3.2 ± 0.4 | 1.3 ± 0.2  (0.9, 1.8) | **<0.0001** |
