# Supplementary material for: Behavioral phenotyping of cancer pain in domesticated cats with naturally occurring squamous cell carcinoma of the tongue: initial validation studies provide evidence for regional and widespread algoplasticity
Source: PeerJ. 2021 Aug 16;9:e11984. doi: 10.7717/peerj.11984 (PMC8375511; doi:10.7717/peerj.11984)
Supplement: Supplemental Information 20 — The statistical analysis of MCPS-FOSCC was performed by Wilcoxon matched-pairs signed rank test; MST and CCT were analyzed by paired t-test. [file peerj-09-11984-s020.docx]

Supplemental **Table S7.** Outcome measure values (Mean ± SD and range) prior to buprenorphine administration (Test 1), after buprenorphine administration (BUP), and the following day (Test 2). The comparison between before and after giving buprenorphine in UFEPS/VET was performed by Wilcoxon matched-pairs signed rank test; MST and CCT were analyzed by paired *t*-test.

| Pain test | Test 1 (Baseline) | BUP | Test 2 | Mean difference between **Test 1 and BUP** (95% CI) | *P*-value | Post hoc power |
| --- | --- | --- | --- | --- | --- | --- |
| UFEPS-FOSCC |  |  |  |  |  |  |
| Total score | 7.8 ± 3.7 | 5 ± 3.3 | 7 ± 3.61 | -2.8 ± 2.6 (-5.6, -0.06) | 0.0938 | 0.73 |
| MST (g) |  |  |  |  |  |  |
| Ipsilateral intermandibular space | 107.4 ± 33.1 | 172.6±59.3 | 114.3 ± 40.0 | 66.9 ± 60.0 (3.9, 129.8) | **0.0412** | 0.76 |
| Contralateral intermandibular space | 104.7 ± 44.7 | 149 ± 47.9 | 96.0 ± 33.2 | 44.3 ± 49.1 (-7.2, 95.8) | 0.0781 | 0.6 |
| Ipsilateral maxilla | 181.6±59.9 | 258.5±126.9 | 167.4 ± 89.0 | 70.3 ± 81.7 (-15.4, 156.1) | 0.0888 | 0.57 |
| Right metacarpus | 225.7±81.8 | 221.8±118.8 | 209.9±67.26 | -51.1 ± 198 (-194.7, 296.9) | 0.5946 | 0.14 |
| CCT (cm) | 3.2 ± 0.4 | 2 ± 0.8 | 2.88±0.48 | -1.17 ± 0.8 (-2.0, -0.3) | **0.0173** | 0.96 |
